# Late combination shows that MEG adds to MRI in classifying MCI versus controls

**DOI:** 10.1016/j.neuroimage.2022.119054

**Published:** 2022-05-15

**Authors:** Delshad Vaghari, Ehsanollah Kabir, Richard N. Henson

**Affiliations:** a MRC Cognition and Brain Sciences Unit, University of Cambridge, UK; b Department of Electrical and Computer Engineering, Tarbiat Modares University, Tehran, Iran; c Department of Psychiatry, University of Cambridge, UK

**Keywords:** Structural MRI, MEG, Multimodal integration, Machine learning, Alzheimer's disease, Mild cognitive impairment

## Abstract

Early detection of Alzheimer's disease (AD) is essential for developing effective treatments. Neuroimaging techniques like Magnetic Resonance Imaging (MRI) have the potential to detect brain changes before symptoms emerge. Structural MRI can detect atrophy related to AD, but it is possible that functional changes are observed even earlier. We therefore examined the potential of Magnetoencephalography (MEG) to detect differences in functional brain activity in people with Mild Cognitive Impairment (MCI) – a state at risk of early AD. We introduce a framework for multimodal combination to ask whether MEG data from a resting-state provides complementary information beyond structural MRI data in the classification of MCI versus controls. More specifically, we used multi-kernel learning of support vector machines to classify 163 MCI cases versus 144 healthy elderly controls from the BioFIND dataset. When using the covariance of planar gradiometer data in the low Gamma range (30–48 Hz), we found that adding a MEG kernel improved classification accuracy above kernels that captured several potential confounds (e.g., age, education, time-of-day, head motion). However, accuracy using MEG alone (68%) was worse than MRI alone (71%). When simply concatenating (normalized) features from MEG and MRI into one kernel (Early combination), there was no advantage of combining MEG with MRI versus MRI alone. When combining kernels of modality-specific features (Intermediate combination), there was an improvement in multimodal classification to 74%. The biggest multimodal improvement however occurred when we combined kernels from the predictions of modality-specific classifiers (Late combination), which achieved 77% accuracy (a reliable improvement in terms of permutation testing). We also explored other MEG features, such as the variance versus covariance of magnetometer versus planar gradiometer data within each of 6 frequency bands (delta, theta, alpha, beta, low gamma, or high gamma), and found that they generally provided complementary information for classification above MRI. We conclude that MEG can improve on the MRI-based classification of MCI.

## Highlights

• Resting-state MEG data improve classification of individuals with MCI versus healthy controls when combined with features from structural MRI.
• “Late” combination of MEG and structural MRI predictions performs best, i.e., better than “Intermediate” combination of feature kernels or “Early” combination of features.
• Classification accuracy with MEG alone never exceeded that for MRI alone.
• While the choice of MEG sensor-type (magnetometers or planar gradiometers) did not make a big difference, the choice of frequency band did.
• Potential confounds in any classification problem can be accommodated more properly using a kernel-based approach.

## 1 Introduction

Alzheimer's disease (AD) is an age-related neurodegenerative disorder and a major challenge for healthcare and social care due to its high prevalence and costs. Early detection of AD is critical for treatment and prevention, and this requires a robust biomarker that can identify the disease in cases such as Mild Cognitive Impairment (MCI) (Petersen, 2009). Such biomarkers may also provide disease progression monitoring in clinical trials. Neuroimaging techniques offer a range of potential biomarkers of structural, metabolic and functional changes in the brain related to AD and MCI (Cabeza et al., 2018; Tartaglia et al., 2011; Woo et al., 2017).

Magnetic Resonance Imaging (MRI) is such a key technique, which can be tuned to various tissue properties. The most common of these is the T1-weighted contrast between gray-matter and white-matter – so-called “structural MRI” (sMRI) – which can be used to estimate the reduction in the gray-matter volume of various brain regions owing to the atrophy in AD. This is a standard approach in the assessment of dementia (Frisoni et al., 2010; Nestor et al., 2004). However, AD affects neuronal physiology before cell death and atrophy (Dubois et al., 2016; Han et al., 2012; Jack et al., 2017, 2013). Although functional MRI (fMRI) can be used to measure changes in physiological activity and/or connectivity (Agosta et al., 2012; Suckling et al., 2015; van den Heuvel and Hulshoff Pol, 2010; Wang et al., 2006), fMRI only provides an indirect measure of neural function. This is because it relies on the haemodynamic response to neural activity, and is therefore confounded by changes in the brain's vasculature that occur with age and neurodegenerative disease (Tsvetanov et al., 2021). Furthermore, the slow haemodynamic response means that fMRI is largely blind to neuronal dynamics above 0.1 Hz.

More direct measures of neural activity can be obtained by Electroencephalography (EEG) and Magnetoencephalography (MEG), which measure the electromagnetic fields produced by dendritic dipoles within active neurons (Hari and Puce, 2017; Stam, 2010). These can be sampled at a resolution of milliseconds, revealing a rich repertoire of neural dynamics, including oscillatory rhythms that occur at frequencies between 2 and 100 Hz, such as “alpha” (8–12 Hz) and “gamma” (30+Hz), some of which have also been implicated in AD (Engels et al., 2017; Giovannetti et al., 2021; López-Sanz et al., 2018; Mandal et al., 2018; Osipova et al., 2005; Stam et al., 2009, 2006; Wang et al., 2017; Wiesman et al., 2021). MEG offers an advantage over EEG in that the magnetic fields are less distorted and smoothed by the brain-skull interface than are electric fields, resulting in higher spatial resolution (Maestú et al., 2019). We, therefore, focus on MEG measures of neural activity (during rest) to see if they provide information for MCI classification that is complementary to the structural information in sMRI.

The general advantage of multimodal integration (combining information from more than one neuroimaging technique) has been appreciated for many years, on the assumption that each modality reveals information about somewhat different aspects of the underlying neural circuity (Engemann et al., 2020; Henson et al., 2011; Kumral et al., 2020; Nentwich et al., 2020; Schouten et al., 2016) and consistent with findings that combining multiple modalities can improve AD classification (Patel et al., 2008; Polikar et al., 2010). For example, using various different machine learning techniques, Patel et al. (2008) and Polikar et al. (2010) have demonstrated that combining EEG and sMRI improved diagnosis of AD versus controls compared to using individual modalities alone. However, the EEG data in these studies were collected during an auditory oddball paradigm, rather than the more common resting-state used here. Colloby et al. (2016) is the only study we could find that combined resting-state EEG and sMRI, but they focused on distinguishing relatively late cases of AD versus Lewy-body dementia, rather than distinguishing MCI versus healthy controls, as done here (a study by Farina et al. 2020).

Neuroimaging produces many measurable properties (or “features”) from each participant, such as ∼100,000 voxels in an sMRI image or ∼1000,000 timepoints in ∼300 sensors in MEG, which normally exceed the number of participants (typically ∼100). Identifying which features are important for classifying AD, therefore, benefits from machine learning techniques (Wolfers et al., 2015), such as kernel-based approaches. A kernel is a square matrix containing a measure of the similarity between every pair of participants in their feature values. Classification based on kernels rather than raw data is robust and efficient for high-dimensional pattern classification (Schölkopf and Smola, 2018; Shawe-Taylor and Cristianini, 2004). Here we used Multiple Kernel Learning (MKL) (Gönen and Alpaydın, 2011), which optimises the weighting of kernels from each modality, which is generally better than simply concatenating features across modalities, particularly when the modalities differ in the number of features and/or those features are incommensurate (such as volume in mm^3^ for sMRI versus magnetic field power in fT^2^ for MEG) (Donini et al., 2016; Hughes et al., 2019; Korolev et al., 2016; Liu et al., 2018; Peng et al., 2019; Wee et al., 2012; Youssofzadeh et al., 2017; Zhang et al., 2011). Adding kernels to the classification model is also a better way to accommodate potentially confounding variables (such as the age of MCI and Control cases) than is the more common approach of first adjusting the data (features) for those variables (Dinga et al., 2020; Snoek et al., 2019).

**Fig. 1:**
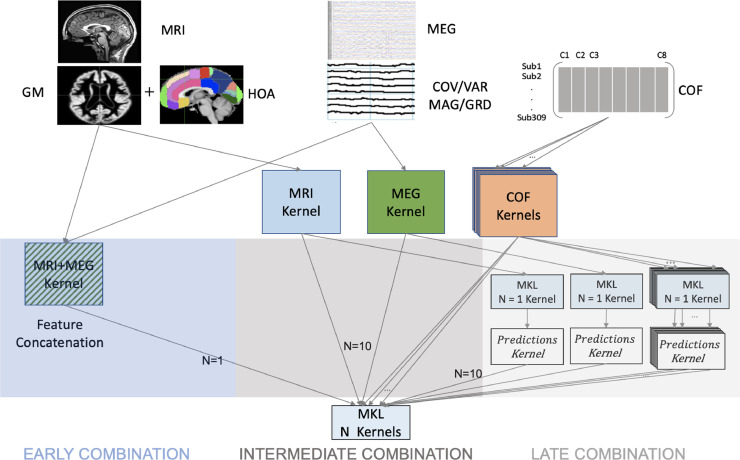
Schematic showing the three different combination stages used here: Early (feature concatenation), Intermediate (kernel combination) and Late (decision combination). N is number of kernels. GM = Gray-Matter, HOA = Harvard-Oxford Atlas, COFs = (potential) Confounds.

Most importantly, we compared results from combining modalities at three different stages: early, intermediate and late (Fig. 1). By Early combination, we refer to the simple concatenation of the features of each modality, after normalizing them by their standard deviation across participants (i.e., to unit-less quantities with comparable numerical range). By Intermediate combination, we refer to the typical MKL approach of optimizing the weighting of kernels derived from the features of each modality (or confound). By Late combination, we refer to the application of MKL to kernels derived from the class predictions after classifiers are run on each modality separately. The latter is closer to the “ensemble learning” philosophy (Kuncheva, 2014) and the “stacking” approach used by Engemann et al. (2020).

## 2 Materials and methods

### 2.1 Participants

We included resting-state MEG and T1-weighted structural MRI data from the BioFIND dataset (Vaghari et al., 2021), which includes individuals with Mild Cognitive Impairment (MCI) and Healthy Elder Controls (HEC) from two sites: the MRC Cognition & Brain Sciences Unit (CBU) in Cambridge, England, and the center for Biomedical Technology (CTB) in Madrid, Spain. The CBU controls were recruited from the CamCAN sample (www.cam-can.org) who are screened to be healthy, i.e., have MMSE (and indeed ACE-R) scores above conventional cut-offs, as well as other screening described in CamCAN paper (Shafto et al., 2014). The CTB controls had a full neuropsychological assessment to confirm normal cognition, and the same type of MRI assessment as that done in the MCI group, i.e., a radiologist reported MRIs as normal.

In general, the MCI diagnosis was determined with the intermediate probability according to the National Institute on Aging–Alzheimer Association criteria (Albert et al., 2011), i.e., given by a clinician based on clinical and cognitive tests, self- and informant-report, and in the absence of full dementia or obvious other causes (e.g., psychiatric). More specifically, for the CBU data, individuals were diagnosed with MCI after referral for symptoms, mainly memory problems (i.e., they were not derived by screening of cognitively asymptomatic people). The diagnosis was made in a regional memory clinic, including ACE/ACER and MMSE tests as standard, with a significant deficit in memory domain tests. PET and fluidic Biomarkers were not used as standard, although all had structural brain imaging (usually MRI in the clinic unless contraindicated, when a CT is occasionally used) and clinical follow-up in support of the diagnosis. By definition of MCI, sufferers had functional independence at the time of diagnosis. sMRI was used to exclude other pathologies and to identify features consistent with MCI/AD pathology (e.g. MTL atrophy without mass lesion, high vascular burden).

For the CTB data, the diagnosis of MCI was based on criteria from Frisoni et al. (2011). It was based on a mixture of clinical and quantitative approaches, but MMSE score was not the sole criterion. The diagnosis required an impairment in the memory domain and/or other cognitive functions, but a "memory complaint" was not required, so some participants showed awareness of their cognitive problems and others did not. Indeed, for CTB data participants were not aware of their current cognitive status. Therefore, they did not have to show cognitive complaints, but memory/cognitive failures were reported by close companions. The only biomarkers used systematically were brain morphology (from MRI) and APOE. As a requirement for the diagnosis criteria, MCI cases were able to still perform their daily living activities. MRI (T1, T2 and/or FLAIR) was used to rule out a vascular disorder, and any other type of neurological disease (i.e., tumor, stroke, infection) that could better explain the cognitive symptoms.

After excluding 15 cases without an MRI, and 2 with MRIs with dental artifacts, there were 163 HEC and 144 MCI datasets. A summary of sample characteristics is reported in Table 1, which includes variables that could affect MCI status (such as education) or could affect brain activity in general (such as time of day of testing) or could affect the MEG data specifically (such as head motion or distance from sensors). A small number of missing values were imputed using the mean from non-missing values for each variable: 5 missing values for MMSE (1.5% of cases, all MCI), 12 missing values for Education (3.7% of cases; 11 MCI, 1 HEC) and 26 missing values for mean and standard deviation of motion (8.0% of cases; 21 MCI and 5 HEC). We used MATLAB's “*knnimpute*”, applied across the whole sample, which uses the value from the nearest neighbor based on Euclidean distance. The imputed data are available in the tab-separated value file “participants-imputed.tsv” on the GitHub repository (https://github.com/delshadv/MRI_MEG_Combination).

**Table 1:** Characteristics of BioFIND participants with clean MRIs. CBU = Cognition & Brain Sciences Unit (Cambridge); CTB = center for Biomedical Technology (Madrid). Translation calculated every second from Maxfilter stage (see Vaghari et al. 2021). Distance from sensors was calculated after coregistering the MEG to the MRI, and averaging the Euclidean distance between every sensor and each of 2562 vertices on a cortical mesh. SD = standard deviation. MMSE = Mini-Mental State Examination.

Data Characteristic		Groups	
		HEC	MCI
1	**Site (CBU/CTB)**	89/74	63/81
2	**Sex (M/F)**	82/81	74/70
3	**Age (years)**	71.2 (7.0)	72.8 (6.8)
4	**Time of Day (24 h)**	12.8 (2.4)	12.6 (2.1)
5	**Mean head translation (mm)**	2.0 (1.8)	2.1 (1.8)
6	**SD head translation (mm)**	1.1 (1.1)	1.2 (1.1)
7	**Distance from sensors (mm)**	113.0 (1.5) x10^3^	113.3 (2.3) x10^3^
8	**Education years**	14.5 (4.4)	11.0 (5.2)
-	**MMSE**	28.8 (1.2)	25.9 (3.4)

### 2.2 Confounds

There were more HEC cases from the CBU site and more MCI cases from the CTB site, but the number of males/females was close to be matched across the two groups. A two-sample T-test confirmed that, on average, the MCI group had lower MMSE scores, as expected from their clinical diagnosis, T(305)=9.63, *p* < 0.001. However, they also had fewer years in education, T(305)=6.41, *p* < 0.001, and were slightly older on average, T(305)=2.09, *p* < 0.05, which may confound any group differences in MRI and/or MEG. While none of the other variables in Table 1 differed significantly between the two groups, T's<1.46, *p* > 0.14, it is possible that combinations of them could predict MCI status above chance. We, therefore, included all of them as potential confounds (COFs), except MMSE. The reason we did not include MMSE as a confound is that this cognitive measure informs the MCI diagnosis, so would be circular (biased) to use as a predictor.

### 2.3 . MEG preprocessing

MEG data were acquired while participants were seated inside a magnetically shielded room (MSR). The CBU MSR is made by Imedco and uses single layer mu-metal plates, while the Madrid MSR is made by Vaccumschmelze and has two layers (for further technical details, see Vaghari et al. (2021).

The raw data were de-noised using signal space separation (SSS) implemented in MaxFilter 2.2.12 (Elekta Neuromag) to suppress environmental noise (Taulu and Kajola, 2005). For more details of max-filtered data and parameters applied to MEG data in BioFIND, please see Vaghari et al. (2021). The max-filtered (and raw) data are available here: https://portal.dementiasplatform.uk/AnalyseData/AnalysisEnvironment.

The max-filtered data were read into using the SPM12 toolbox (http://www.fil.ion.ucl.ac.uk/spm/; Penny et al., 2006). The minimum duration of resting-state data across participants was 120 s, so the first two minutes of data were used for all participants. A recent study by Wiesman et al. (2021) showed that spectral properties of intrinsic brain activity can be robustly estimated from such short segments of resting-state data. (Indeed, when we repeated the current analyses with 3 min of data, excluding the five participants who had less than 3 min of data, the results were virtually identical). The precise pre-processing steps are provided in the *preproc_meg.m* script in the GitHub repository (see above link). In brief, the 120 s of data was down-sampled to 500 Hz and band-passed filtered from 0.5 to 98 Hz (via a high-pass filter followed by a low-pass filter). The filter type is Butterworth IIR filter with the order of 5 using *spm_eeg_filter.m* function. The continuous data were then epoched into 2s segments, and bad epochs were marked using the OSL automatic artefact detection (https://ohba-analysis.github.io/osl-docs/). The number of bad epochs (*M* = 4.66 for MCI and *M* = 4.03 for HEC, out of 60 total) did not differ significantly between groups, T(305)=1.59, *p* = 0.11. Non-bad epochs were then concatenated again and bandpass filtered within each of six frequency bands.

Unfortunately, not all participants had EOG or ECG recorded, in order to provide objective measures of artifacts. However, note that eyes were closed in all cases, which will abolish blinks and minimize eye motion, while the dominant power in ECG is typically around 1 Hz, which is below the lowest frequency analyzed here. Moreover, the first author conducted a brief visual inspection of data from all participants. This revealed residual some spikes, particularly in the high-frequency bands (low gamma and high gamma). After band-pass filtering, these spikes were detected and corrected using MATLAB's *filloutliers* function (using the 'median' option with a 'ThresholdFactor' of 3 for detection, and the “clip” method for replacement). The number of such spikes (outliers) did not differ significantly between groups in any frequency band, T's<1.33, *p*’s > 0.19, except Delta, T(305)=2.60, *p* = 0.01, and was always fewer than 1% of samples.

### 2.4 MRI preprocessing and feature extraction

The de-faced, T1-weighted scans were processed in SPM's DARTEL-VBM pipeline (Ashburner and Friston, 2000), as implemented in the *preproc_mri.m* script in the GitHub repository. Each MRI was first segmented into gray matter (GM) and white matter (WM) probability maps. These GM and WM images were then warped to an average template for the sample using diffeomorphic warping in the DARTEL toolbox (Ashburner, 2007), and this template transformed to MNI space. These transformation parameters were then applied to each participant's GM image, modulated so as to preserve local GM volume, and the GM values resampled into MNI space together with a spatial smoothing by a 1 mm FWHM isotropic Gaussian kernel to remove interpolation artifacts. Finally, 110 MRI features were used for classification, representing the mean across voxels within the 110 anatomical ROIs of the Harvard-Oxford Atlas (Kennedy et al., 1998; Makris et al., 1999).^1^ In case the HOA atlas is too coarse to reveal anatomical changes in MCI, Supplementary Section S6 shows results using GM volume in all 390,189 voxels as features instead.

### 2.5 MEG feature extraction

We focused on sensor level features, rather than reconstructing the sources of the MEG data. Firstly, it is unclear whether source reconstruction provides additional information for classification with real data. Since sensor data are a linear combination of source data (mixed through a “forward model”, Hari and Puce, 2017), there no degrees of freedom is gained when estimating source amplitudes. Having said this, when classifying on the basis of non-linear functions of the data, such as the (co)variance (power) features used here, this equivalence between sensor and source features is lost (Sabbagh et al., 2019). It is true that an accurate forward model (which accommodates differences in head position and anatomy) helps align features across participants, and simulations show improvements when estimating sources (Sabbagh et al., 2020), though in practice there are always errors in the forward model and noise in the data that prevent perfect alignment. Indeed, there are normally more sources than sensors, rendering the source estimation problem ill-posed, and requiring additional assumptions to regularize the solution. Secondly, and perhaps more importantly, since an sMRI is necessary to construct an accurate head model, for the present purposes of comparing MRI and MEG classification, we did not want information from the MRI to contaminate the MEG features.^2^

The MEG data come from one magnetometer (MAG) and two, orthogonal planar gradiometers (GRD) at each of 102 locations above the head (i.e., 306 channels in total). MAGs measure the component of the magnetic field that is perpendicular to the sensor, whereas GRDs estimate the spatial derivative of the magnetic fields in two orthogonal directions in the plane of the sensor (which is roughly parallel to the scalp). By taking the spatial derivative, GRDs are less sensitive to distant sources, such as environmental noise, and so have a higher signal-to-noise ratio for signals close by, i.e., in the superficial cortex. By contrast, MAGs are more sensitive to deeper signals in the brain, but also more susceptible to noise. The best way to combine GRD and MAG data is a matter of contention because they have different SI units (Garcés et al., 2017), but by using separate kernels for each, we do not need to combine them directly.

We focused on the second-order moments of MEG data, i.e., the data covariance across sensors (which is incidentally what most source localization methods are based on). The variances are related to the signal power in each sensor, whereas the covariances are related to the cross-spectral power. Note that sensor covariances capture aspects of both brain activity and connectivity (and can only be attributed solely to connectivity between sources after adjusting for field spread, i.e., linear mixing by the forward model, Engemann et al., 2020). This meant 102 features for MAG variance and 204 features for GRD variance, both corresponding to the spatial distribution of power across the scalp, with 5151 covariance features for MAGs and 20,706 covariance features for GRDs. Note however that our preprocessing of the MEG data (during the MaxFilter stage above) includes a dimension reduction to 69 and 66 components for MAG and GRD respectively, so the rank of the covariance matrices is less. This does not matter for our classification results, since when we reduced the dimensionality further, using principal component analysis (PCA) (Wold et al., 1987) to calculate the number of components needed to explain 95% of the total variance in the MEG features across participants, the pattern of classification results was hardly changed (see Supplementary Section S4). Note that, to avoid bias, PCA was applied in the training set and the projection was then applied to the test set (see *mkl_ens_nestedcv.m* and *mkl_ens_n.m* in the paper's GitHub repository).

Each of these features was calculated for 6 frequency bands: Delta [2–4 Hz], Theta [4–8 Hz], Alpha [8–12 Hz], Beta [12–30 Hz], low Gamma [30–48 Hz] and high Gamma [52–86 Hz]. As described in preprocessing section, the frequency transform is applied to continuous data (without epoching). Note that we did not relativize power in each frequency band to the total power (across all frequencies), e.g. by normalizing the time-series before estimating power (Hughes et al., 2019). While such normalization allows for differences in overall signal strength owing to the proximity of the head (brain) to the sensors, the danger of normalizing power in this way is that it could also remove true power differences between MCI and HEC. We, therefore, used absolute power, but by including the mean distance between the brain and sensors as a confound, were able to make some allowance for different head positions (after squaring, since the magnetic field strength falls off with at least the square of distance).

### 2.6 Multimodal classification

We compared three stages of combining MEG and MRI data: 1. Early combination: features from MRI and MEG are normalized and then concatenated and fed to a single classifier; 2. Intermediate combination: features from MRI and MEG are projected to kernels and MKL then forms an optimal kernel from a function of the original kernels that maximises multimodal classification accuracy; 3. Late combination: MRI and MEG features are fed to one or more classifiers whose continuous-valued outputs (a prediction related to the probability of membership of each class or distance to decision boundary) are then combined in an optimal way, again using MKL (Gönen and Alpaydın, 2011; Kuncheva, 2014; Noble, 2004). According to a common taxonomy of classifier ensemble methods, our Late combination is a type of “stacked generalization”, since the outputs of the individual classifiers for each modality are treated as inputs to a meta classifier (here EasyMKL) (Engemann et al., 2020; Wolpert, 1992). It is also worth mentioning that in pattern recognition, Early combination is categorized as “feature level” combination whereas Late combination is classified as “decision level” combination. Using Intermediate or Late combinations can be a natural way to control the confounds (COF) effect, namely by adding one or more kernels (see Discussion). A comparison schematic of these approaches is presented in Fig. 1.

Kernel methods project the data features into matrices (kernels) that represent the similarity of the feature vectors between every pair of observations (here, participants), and optimize classification performance based on these kernels (thus each kernel in the present case was a 307×307 matrix, regardless of the number of features per modality). Kernels are the basis of several types of classifiers such as Support Vector Machines (SVM) (Cortes and Vapnik, 1995) and MKL (Gönen and Alpaydın, 2011). Let {$x_{i},y_{i}$}^L^ be the training set, where *i = 1,2,…L* is the number of training examples. Then, a linear MKL learns a coefficient vector η that weights each kernel ***k*** to produce an optimal kernel ***K*** according to:

$$K\left(x,x^{\prime }\right)=\sum_{n=1}^{N}\eta_{n}k_{n}\left(x,x^{\prime }\right)$$

$$\text{with}\;\sum_{n=1}^{N}\eta_{n}=1,\;\;\eta_{n}\geq 0$$

where *n = 1,2,…N* is the number of base kernels. Note that we used linear kernels (as well as a linear combination), which is recommended when the number of features is larger than the number of participants, i.e., when there is insufficient data to fit more complex nonlinear kernels. The decision function of an MKL problem which is the distance to the decision boundary for classification models can be then expressed in the form:

$$f\left(x\right)=\sum_{i=1}^{L}\alpha_{i}^{*}\;K\left(x,x_{i}\right)+b^{*}$$

Where $\alpha_{i}^{*}\;$and $b^{*}$ are some coefficients to be learned from training examples. Learning these coefficients as well as $\eta_{n}$ in a single optimization problem is known as the MKL problem. EasyMKL^3^ is an MKL algorithm that estimates the relative weighting of each kernel by solving a quadratic optimization problem. Kernels that are not helpful for classification are down-weighted. EasyMKL's empirical effectiveness has been demonstrated across a large range of kernel numbers (Aiolli and Donini, 2015; Donini et al., 2016). All features were Z-scored across participants before projecting into kernels, separately for the training set and test set. We also employed L1 (min-max) normalization of kernels to project all their elements to the interval [0 1]. To ensure that any differences between combination methods did not reflect details of the classifier, the same EasyMKL algorithm was used in all cases (even when only *N* *=* *1* kernel in the case of Early combination).

The EasyMKL algorithm uses a regularization parameter, lambda (λ). A value of λ close to 1 penalizes less informative data, though potentially under-fits data, while a value of λ close to zero does not penalize less informative data, so potentially over-fits data (in that results may be sensitive to outliers in the training data;(Hastie et al., 2009). Here, we used (stacked) nested cross-validation (Varoquaux et al., 2017) by grid search with the same scoring as used for evaluation of the model performance to optimize λ, as implemented in the *mkl_ens_nestedcv.m* script in the GitHub repository. This function tunes λ for both the first stage of feature kernel combination and the second stage of decision combination. The value of λ for all analyses are reported in Supplementary Section S8.

Classification performance was estimated using 5-fold cross-validation. Note this applied to both stages of Late combination, i.e., performance was always assessed using the untrained fold. Though the overall sample was unbalanced (with more HEC cases than MCI cases), the training set was always selected to be balanced, and the excess HEC participants were assigned to the test set. Given this imbalance in the test set, we report “balanced” classification accuracy, i.e., the mean accuracy across each class separately. Noise simulations in the Supplementary Section S1 confirmed that our estimation procedure was unbiased.

Cross-validation was repeated 1000 times with random selections of the data, in order to estimate classification reliability and to attenuate bias due to the actual order of the data which is recommended by Varoquaux et al. (2017). Note that the specific random assignment of participants to training/test sets was matched when comparing different combination methods, meaning that classification accuracies could be directly subtracted for each comparison of interest, such that we could determine the percentage of the distribution of differences in classification accuracies that was greater than zero. This provides an approximation of the reliability of any improvement offered by one approach versus another (e.g., combined MEG and MRI versus MRI alone, or Intermediate vs Late combination of MEG and MRI).

### 2.7 Analyses

Firstly, we evaluate classification performance of each modality i.e., MRI and MEG, separately, as well as from the confounding variables. We then combine (via Late combination) the confounding variables with either MRI or MEG, to test whether either imaging modality allows a reliable improvement relative to confounding variables alone. Secondly, we compare Early, Intermediate and Late combination of MRI and MEG modalities. Note that we start with one of MEG features i.e., the covariance of gradiometers in low gamma, but go on to consider other MEG features.

In supporting analyses, Section S1 of Supplementary material uses simulations to compare performance with different sets of signal and noise kernels, to evaluate how well the MKL approach works. In Supplementary Section S2, we repeat the first analyses in the main text for Early and Intermediate combinations, for completeness. In Supplementary Section S3, we try PCA as one of the common feature reduction methods, to see whether the classifier can take advantage of dimensionality reduction. In Supplementary Section S4, we show the classification accuracies for different types of MEG features alone, as well as a comparison of gradiometers versus magnetometers, and covariance versus variance. In Supplementary Section S5, we replace SVMs in the first stage of our Late combination with K-nearest neighbors, random forest, or multi-layer neural networks. In Supplementary Section S6, we use gray-matter volume in all voxels of the MRI image (instead of ROI data) for combining with MEG. Finally, in Supplementary Section S7, we combine MRI and MEG features for classification of the subset of MCI converters versus non-converters.

## 3 Results

### 3.1 Confounds and single MRI and MEG modalities

**Fig. 2:**
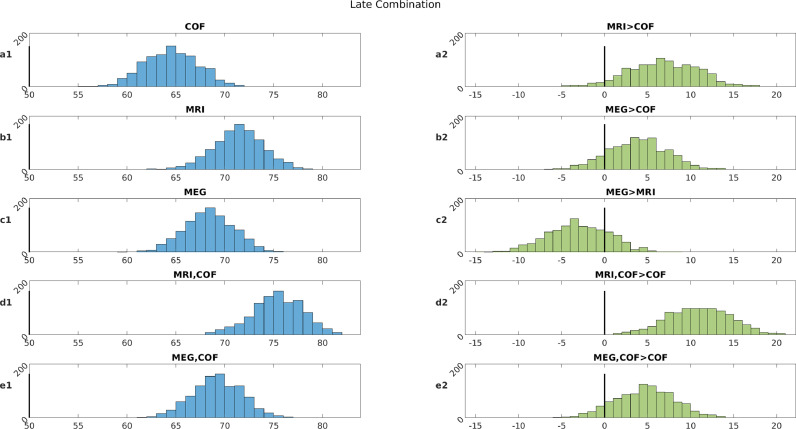
Left column: Classification accuracies (chance = 50%) from 1000 random permutations using Late combinations of MRI, MEG (covariance of gradiometers in low-gamma band) and the 8 potential confounding variables. Right column: Differences in classification performance for each permutation when comparing various combinations of features in left column (where 0 = means no difference). “A,B” means combining two (or nine - in presence of confounds) predictions derived from models trained using modality-type A and modality-type B.

Panel a1 in Fig. 2 shows the distribution across 1000 permutations of classification accuracies based on 8 kernels, each representing one of the potentially-confounding variables (COFs). The mean accuracy was 64.5%, and above chance (50%) on 100% of occasions. These results come from Late combination of the 8 kernels; results using Intermediate and Early combination are shown in Supplementary Section S2. This demonstrates that the two groups (MCI and Controls) were not perfectly matched in these potential confounds, but the reliable above-chance classification (which may be accidental or causal) provides a baseline to compare with classification using the MEG and MRI features.

For a single kernel based on the MRI features (of GM volume within 110 anatomical ROIs), Panel b1 of Fig. 2 shows a mean accuracy of 71.4% (note that there is no distinction between Early, Intermediate and Late combination for a single kernel). To test whether this is a reliable improvement relative to COFs alone, Panel a2 of Fig. 2 shows the distribution of differences between accuracies based on MRI versus COFs (when using the same permutations), which showed that MRI was more accurate on 95.5% of occasions. This demonstrates that MRI provides more information about MCI status than the potential other confounds considered.

For the MEG features, we start with the covariance across GRDs in the low Gamma range (see later for results using other MEG features). Panel c1 of Fig. 2 shows a mean accuracy of 68.4%. While this MEG performance was higher than the baseline provided by the COFs on 86.6% of occasions (Panel b2), it was lower than for MRI on 78.5% of occasions (Panel c2). This finding that MRI is generally better than MEG is not surprising, since an MRI is also often used to define the MCI label, i.e., is likely to be biased (see Discussion). The more interesting comparison is whether combining MEG and MRI improves classification relative to MRI alone, which we return to after the next section.

### 3.2 Adjusting for confounds

Panels d1 and e1 show results from Late combination of the COF kernels and either the MRI or MEG kernel, respectively. The addition of the COF kernels improves accuracy for both neuroimaging variables, but the more important result is in Panels d2 and e2, which show that these combinations are better than COFs alone on 99.7% and 90.7% of occasions for MRI and MEG respectively. This method of combining predictions from confounds (covariates) with those from features of interest (e.g. MRI) is arguably a better way to adjust for confounds than projecting them out of the features of interest themselves (Dinga et al., 2020; Snoek et al., 2019).

### 3.3 Advantages of early, intermediate and late combination of MRI and MEG

**Fig. 3.**
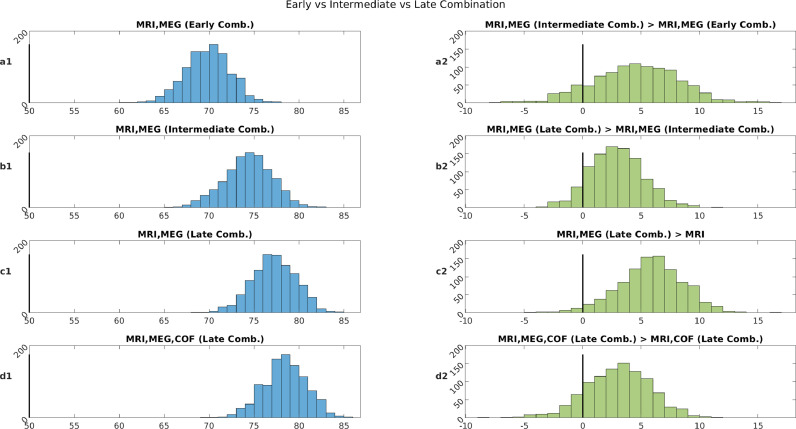
Left column: Classification accuracies (chance = 50%) from 1000 random permutations using MEG, MRI with Early, Intermediate and Late combinations (see methods). Right column: Differences in classification performance for each permutation when comparing various combinations approach (where 0 = means no difference).

Fig. 3 shows classification accuracies when combining the MRI and MEG features at either early, intermediate or late stages. Panel a1 shows that accuracy for early combination is 69.8% which is less than MRI alone (71.4%). This demonstrates that concatenating features from different modalities is not an efficient way to combine them. For Intermediate combination, on the other hand, performance is improved on 87.4% of occasions relative to Early combination (Panel a2), with a mean accuracy of 74.3% (Panel b1). This demonstrates that combining feature kernels is better than concatenating features for these data. Late combination improves performance still further, improving on Intermediate combination on 90.6% of occasions (Panel b2), with a mean accuracy of 77.2% (Panel c1). This demonstrates that combining decision kernels is better than combining feature kernels for these data.

The most important result is shown in Panel c2, which shows that Late combination of MEG and MRI improves classification accuracy compared to MRI alone on nearly 97.5% of occasions. This suggests that MEG provides information about MCI status that is complementary to that in MRI. A similar improvement occurred on 86.8% of occasions when the 8 COF kernels were also added (Panel d1 and d2), showing that this complementary information is also different from anything captured by the potential confounds.

The same general improvement when adding MEG to MRI was also found when massively reducing the ratio of MEG features relative to MRI features by using PCA for feature selection (Supplementary Section S4), or dramatically increasing the number of MRI features by using voxels rather than ROIs (Supplementary Section S6). Similar improvements of multimodal integration were also found when using some popular classifiers other than SVMs (viz. KNN, random forest or neural networks), particularly for those classifiers that did better overall (Supplementary Section S5).

### 3.4 Combining MRI and other MEG features

So far, we selected the COV of GRD in low Gamma as the MEG features, but it is possible that the above results are biased by this selection: i.e., when examining all 24 possibilities of variance/covariance (VAR/COV), gradiometers/magnetometers (GRD/MAG) and the 6 frequency bands, it is possible that one or more of them would show better performance when combined with MRI (than MRI alone) due to chance alone. We, therefore, repeated the above analyses across all frequency bands using both VAR and COV of both GRD and MAG. The results are shown in Table 2, where the top numbers are the mean and standard deviation of classification performance (using Late combination of that MEG feature with MRI), the middle number shows the percentage of permutations in which this combination was better than MRI alone, and the bottom number shows the mean difference between MEG-MRI combination and MRI alone (see Supplementary Section S3 for raw performances for MEG alone).

**Table 2:** Exploring the MEG feature space. The top numbers show mean (and SD in brackets) of classification accuracy when combining the relevant MEG feature with MRI; the middle number shows the percentage of permutations in which this accuracy exceeded that of MRI alone (71.4%), where chance = 50%; the bottom number shows the average difference between MEG-MRI combination and MRI alone.

MEG FeatureFrequency band	COV of MAG	VAR of MAG	COV of GRD	VAR of GRD
Delta2–4 Hz	71.7 (2.6)58.1%+0.22	71.6 (2.5)43.9%+0.10	72.1 (2.5)62.0%+0.51	71.8 (2.5)60.1%+0.29
Theta4–8 Hz	71.5 (2.7)48.7%+0.05	71.6 (2.5)35.5%+0.02	71.6 (2.6)50.1%+0.03	71.5 (2.5)44.5%−0.03
Alpha8–12 Hz	72.0 (2.6)68.5%+0.52	71.7 (2.5)58.4%+0.19	71.3 (2.4)42.2%−0.28	71.0 (2.5)30.5%−0.57
Beta12–30 Hz	73.3 (2.6)90.5%+1.80	71.9 (2.5)69.7%+0.36	74.0 (2.5)84.0%+2.44	73.0 (2.5)86.8%+1.46
Low-Gamma30–48 Hz	76.3 (2.5)96.9%+4.80	74.3 (2.5)94.2%+2.70	77.3 (2.5)97.4%+5.74	75.6 (2.5)96.2%+4.07
High-Gamma52–86 Hz	76.1 (2.5)98.8%+4.60	74.9 (2.5)96.7%+2.46	75.5 (2.5)92.3%+3.96	74.6 (2.5)91.4%+3.01

Note that for low frequencies (Delta, Theta and Alpha), accuracy when combining them with MRI did not consistently exceed that for MRI alone (71.4%) – i.e. was better on around 50% occasions, as would be expected by chance. In fact, adding MEG sometimes reduced classification accuracy, which could either reflect estimation noise, or the fact that the MKL algorithm is not perfect at ignoring kernels that do not improve classification (see simulations in Supplementary Section S1).

For the Beta band, combined accuracy increased slightly, being better than MRI alone on 84% of occasions for the COV of GRD. For low Gamma, multimodal accuracy was consistently better for all four types of feature, with COV doing better than VAR/COV, and GRD doing better than MAG (see Supplementary Section S3 for more details). The same was also true of high Gamma. The overall finding that multimodal combination improved accuracy for over a third of the feature space suggests that the improvement is not a fluke occurrence.

## 4 Discussion

The main finding of the present study was that certain features of MEG resting-state data, particularly in the low and high Gamma frequency range, improve the classification of individuals with MCI versus healthy controls when combined with features from structural MRI data. While classification accuracy with MEG alone never exceeded that for MRI alone, this is not necessarily surprising, since an MRI is typically used by the clinicians to support the diagnosis of MCI (i.e., giving MRI an unfair advantage). The important result was that combining MEG with MRI improved classification relative to MRI alone. This indicates that MEG contains complementary information about MCI. This information might include changes in functional activity and/or connectivity that precede structural change, at least as measured by regional gray-matter volume as here (Dubois et al., 2016; Han et al., 2012; Jack et al., 2017, 2013).

The second main finding of the present study was that Late combination of MEG and MRI data was best for multimodal classification, i.e., better than Intermediate or Early combination. Late combination here refers to combining the class predictions of classifiers trained on each modality separately, analogous to ensemble learning (Kuncheva, 2014). Permutation tests showed that this combination at the “decision-level” reliably improved accuracy relative to Intermediate combination at the “feature-level”, where kernels derived from the features of each modality were combined directly. As expected, Intermediate combination via kernels was in turn better than simply concatenating (normalized) features into a single kernel. Note that these findings were obtained when the same classification algorithm (EasyMKL; (Aiolli and Donini, 2015) - i.e., multi-kernel learning of support vector machines - was used for each level of multimodal combination.

A third finding was that the covariance (COV) of the planar gradiometers (GRD) generally provided the best MEG features, and it was important to consider high-frequency components of the MEG data, specifically the low (30–48 Hz) or high (52–86 Hz) Gamma range. Accuracy when using the Beta range did not produce such a large improvement over MRI alone, while that for Alpha, Theta or Delta provided no improvement. This suggests that the important information for MCI classification exists in frequencies above approximately 30 Hz (see below for further discussion).

A fourth outcome was the demonstration that potential confounds in any classification problem can be accommodated within a multimodal approach, in which confounds are combined with the features of interest during classification (which applies whether the combination is Early, Intermediate or Late). To “control for” such confounds, one can show that classification accuracy with the combined data reliably exceeds that for the confounds alone. This is better than the common approach of first adjusting the features of interest by the confounds (e.g., by projecting out of the features of interest anything that can be explained by a linear combination of the confounds, i.e., before creating the feature kernels), because it takes into account the potentially shared dependency between the features of interest and the confounds in their ability to predict the class (Dinga et al., 2020). Furthermore, some classifiers are non-linear, and therefore potentially sensitive to effects of confounds that cannot be removed from the features by linear methods.

### 4.1 Complementary information in MEG/EEG for MCI classification

Previous studies have shown that EEG and/or MEG provide complementary information beyond structural MRI, whether that be in predicting age (Engemann et al., 2020), or classifying types of dementia (Colloby et al., 2016; Patel et al., 2008; Polikar et al., 2010). Some of these have used evoked EEG responses during tasks (e.g., auditory oddballs Patel et al. 2008, Polikar et al. 2010), which may provide further information on neurodegeneration than the resting-state data used here, though resting-state data are more common and easier to obtain, particularly in patients who might struggle with some tasks. In the data paper describing the BioFIND dataset (Vaghari et al., 2021), we reported validation analyses that showed that MEG power across all sensors, or power across all cortical sources, or connectivity between all pairs of sources (based the correlation of the power envelopes) achieved similar classifcation accuracies between 63 and 67%, comparable to the figure of 68% here for MEG alone (at least for COV of GRD in low Gamma). However, in that paper, we did not compare MEG classification with that from MRI. In a previous paper describing the smaller BioFIND project (Hughes et al., 2019), we did report preliminary findings that Intermediate combination of MEG and sMRI improves classification, using a subset of roughly half (*N* = 168) of the present sample (that was available at that time). However, this finding used different features (interpolated 3D scalp-frequency power images for MEG and voxel-level GM images for sMRI) and did not establish the reliability of the improvement using permutation. The present work confirms the reliability of these findings and extends the approach to demonstrate the added value of Late combination of modalities, and the potential value of covariance, rather than power, across MEG sensors within certain frequency bands.

It is important to note that we have only used one type of structural brain information – namely gray-matter volume as estimated from a T1-weighted MRI. Other MRI modalities (e.g., T2-weighted MRI, diffusion-weighted MRI, magnetic resonance spectroscopy, MRS) – or indeed even other features from the current T1-weighted images, such as cortical thickness – might enable better MCI classification, to the extent that MEG no longer adds further improvement. Furthermore, other imaging techniques like PET might do better still, given their ability to measure neurotransmitters or molecular pathologies directly related to AD. However, our main purpose here was to compare MEG with the most common type of brain image available on individuals with MCI, and the most common type of informal inspection done by clinicians, i.e., looking for gray-matter atrophy.

We cannot tell whether the complementary information provided by MEG here relates to the fact that it measures brain function rather than brain structure, or that it provides a measurement of brain function with different spatial and temporal properties than other functional techniques like fMRI. Indeed, it would be interesting to apply the present multimodal classification approach to fMRI data, to see if MEG continues to provide more information than fMRI. If MEG does not improve classification beyond fMRI, then the key additional information for better MCI classification might simply be the inclusion of measures of brain function rather than structure; alternatively, if MEG does improve beyond fMRI, it may be that MEG captures neural activity more directly (bypassing the vascular confounds in fMRI) and/or neural activity that is beyond the temporal resolution of fMRI. Unfortunately, resting-state fMRI is not available in the BioFIND dataset, but is likely to be available together with MEG/EEG and sMRI in future cohorts being studied around the world. Likewise, one could test whether the improved spatial resolution of MEG over EEG also provides additional information for MCI classification and whether task-based MEG/EEG provides additional information beyond the resting-state data used here.

Finally, we cannot tell whether MEG provides complementary information beyond neuropsychological tests. The only cognitive test available on all participants in the current dataset is the MMSE, which is a very brief screening instrument. We did not compare classification with MEG to that with MMSE because the latter was one of the main determinants of the MCI diagnosis (even more so than MRI). It is possible that other more detailed cognitive assessments (not used in patient diagnosis) would be as, or more, effective than brain measures from MEG or MRI in detecting AD and predicting long-term outcome.

### 4.2 Multimodal classification approaches

Our best classification accuracy of 77% when using MEG and MRI may not seem particularly impressive, for example, relative to figures reported in other papers using different modalities and datasets. We did explore some other common classifiers (such as KNN, random forest and multi-layer neural networks), which gave similar results (Supplementary Section S5). However, it is important to note that our aim was not simply to achieve the best classification possible. For example, while we found similar results after PCA to reduce the feature dimensionality (Supplementary Section S4), we could have employed more sophisticated feature selection approaches that might have improved classification accuracy, particularly given the large number of MEG features relative to participants, or we could have tried to minimize effects of field spread on our second-order (covariance) features by employing spatial filtering or Riemannian Embedding (Sabbagh et al., 2020). Furthermore, we could have used neuroscientific knowledge to select features, for example, based on the knowledge that the medial temporal lobes include some of the structures affected in the earliest stages of AD (Frisoni et al., 2010; Shi et al., 2009). Rather, our aim was only to compare the relative performance of different modalities and different methods of combining those modalities, while holding other factors constant.

It is also important to note that the “MCI” label typically captures a range of aetiologies, of which neurodegeneration, and specifically AD, is only one. For example, some of the participants labeled as MCI in the BioFIND dataset may in fact have healthy brain structure and/or function (i.e., no evidence of early AD), but just perform poorly on cognitive tests because of other reasons like depression. Indeed, an appreciable proportion of clinically diagnosed MCI cases later turn out to have no detectable AD pathology (Petersen, 2009). Conversely, some participants labeled as healthy controls may have had early AD and impairments of brain function and/or structure, but performed normally on cognitive tests because of high pre-morbid ability or some form of “cognitive reserve” (Stern, 2009). This would mean it is difficult for any classifier to identify a consistent set of features (in this heterogeneous group) that perfectly predicts our two classes. These issues can only be resolved by longitudinal follow-up, possibly with additional biomarkers (e.g., CSF Tau levels) and ultimately post mortem examination to confirm who had AD. While we did analyze the follow-up data that are available on a subset of the participants labeled MCI in BioFIND (see Supplementary Section S7), the numbers are relatively small (∼50 converters and ∼50 non-converters), and further follow-up is needed, as well as additions from other datasets with larger numbers of cases (that are accessible to researchers, like BioFIND).

It is important to note that, while the BioFIND dataset may be the largest sample of individuals with MCI and controls with MEG data, it is still small for machine-learning approaches, relative to the potential number of MEG features. This dearth of training data may also explain why higher classification accuracies have been reported for other neuroimaging markers (e.g., sMRI) for which larger databases exist, such as ADNI (http://adni.loni.usc.edu/about/). As a reference, using MRI alone on the ADNI database, Liu et al. (2020) reported an accuracy of 76% for classifying MCI cases versus healthy controls, using sophisticated deep neural net classifiers. Interestingly, this is only ∼4% more than achieved here using standard SVMs on a smaller set of MRI cases. However, the convolutional neural network architecture described can be considered for future studies focusing on the best classification performance where for example, sMRI features can be extracted using convolutional layers.

Furthermore, in situations with many more features than cases, overfitting is likely (Cawley and Talbot, 2007; Cristianini and Shawe-Taylor, 2000; Han and Jiang, 2014). This is a situation where Late combination might increase generalization to new datasets, by virtue of the combination of decisions being more robust to over-fitting (Kuncheva, 2014; Wolpert, 1992). Indeed, the simulations in Panel d of Supplementary Section S1 confirm that Late combination can be better than Intermediate combination when more noise features are added, i.e., is more robust against the addition of weak (in terms of accuracy) classifiers trained on noisy features. Note however that Late combination is not always better than Intermediate combination, and multi-kernel combination is not always better than simple feature concatenation (Early combination), as can be seen for the 8 COFs in Supplementary Section S2, i.e., in situations with relatively low numbers of features.

### 4.3 Optimal MEG features for MCI classification

Our finding that low and high Gamma frequencies provide the information that is complementary to MRI is consistent with some previous M/EEG studies of MCI or genetic risk that highlight the importance of the gamma band (Bajo et al., 2010; Luppi et al., 2020; Missonnier et al., 2010; van Deursen et al., 2008) (Koelewijn et al., 2019). However, differences (e.g., in source level power and/or connectivity) were more often found at lower frequencies. For example Garcés et al. (2013), Hughes et al. (2019), López et al. (2014), López-Sanz et al. (2018), Maestú et al. (2019), Nakamura et al. (2018) have argued that the alpha band is best for distinguishing MCI versus controls. One possibility is that the information about MCI status provided by Alpha power is correlated with the gray-matter atrophy provided by MRI, which is why we did not find any improvement when combining MRI with MEG covariance in this frequency band. However, it is worth noting that the best classification accuracy with Alpha alone (∼59%) was still considerably lower than for Gamma alone (∼68%; Supplementary Section S3). Another reason for this discrepancy in the literature may reflect the features used, for example, the amplitude or frequency of the prominent Alpha peak in MEG and EEG power spectra is a type of feature that is not simply (e.g. linearly) derivable from the Alpha covariance matrix used here. Other discrepancies may owe to the use of different and relatively small samples (*N* < 100), possibly with different definitions of individuals with MCI, and to the wide range of methodological approaches (Yang et al., 2019). Indeed, when we examined the classification of converters versus non-converters within the MCI group only, we found a trend for lower frequencies like Delta and Theta to provide more complementary information than the Gamma that was optimal for classifying the MCI versus Control groups (Supplementary Section S7). Thus, we do not wish to claim that Gamma frequencies, or more specifically covariance of planar gradiometers, are always the best MEG features to use for early detection of AD. We only used the sensor covariance matrix as a simple but inclusive measure of functional activity and connectivity and explored the range of frequency bands because of prior evidence that frequency matters. It is worth mentioning that there was some evidence that covariance classified better than variance (Supplementary Section S3), which may be because the sensor covariance, though not a pure measure of brain connectivity, might capture more aspects of connectivity than sensor variance. Nonetheless, future studies could use the present framework to ask whether specific MEG features offer additional information about MCI over other MEG features.

## Code availability

The custom written codes to implement all validation analyses is available on GitHub (https://github.com/delshadv/MRI_MEG_Combination). All MEG and MRI features as well as other derived variables are available in comma-separated value (.csv) files in the “derived” directory within the repository. The raw data are available on the DPUK website (https://portal.dementiasplatform.uk/Apply) cited in the main paper.

## Author statement

We freely provide the codes in the GitHub repository i.e. https://github.com/delshadv for the kernel-based approach, as well as for the MRI and MEG pre-processing and feature extraction steps while the raw data are available on request from the Dementia Platform UK (DPUK). Necessary links to access data are available in the main text. Furthermore, all features that are necessary to reproduce main results like MEG variance, MEG covariance and MRI ROI data are available on both paper's GitHub and DPUK portal in case one does not have the interest or time to reproduce the preprocessing steps.

## CRediT authorship contribution statement

**Delshad Vaghari:** Conceptualization, Formal analysis, Writing – original draft. **Ehsanollah Kabir:** Writing – original draft. **Richard N. Henson:** Conceptualization, Writing – original draft.

## Declaration of Competing Interest

None declared.

## Appendix Supplementary materials

Image, application 1

## Footnotes

1 ROI numbers 111–116 in the HOA are spurious or non-cortical.

2 There are data-driven alternatives, for example, to adjust the sensor covariance to more accurately reflect source covariance using Riemannian Embedding (Sabbagh et al., 2020), which could be tried in future and compared with present results.

3 For more theoretical view including mathematical formulation of EasyMKL see (Aiolli and Donini, 2015) Section 4.
