# Supplementary material for: Late combination shows that MEG adds to MRI in classifying MCI versus controls
Source: Neuroimage. 2022 May 15;252:119054. doi: 10.1016/j.neuroimage.2022.119054 (PMC8987738; doi:10.1016/j.neuroimage.2022.119054)
Supplement: Supplementary file 1 [file mmc1.docx]

# Supplementary Material for “Late Combination shows that MEG adds to MRI in classifying MCI versus Controls”

Delshad Vaghari, Ehsanollah Kabir & Richard N. Henson

## S1. Noise Simulations

We conducted a number of simulations to check the validity of our multimodal combination algorithm and cross-validation scheme. We used the same MCI/HEC labels as the real data in the main paper, i.e., 307 observations. This is performed by the MATLAB script *noise_sim.m* in the GitHub repository, using fixed hyperparameters of 0.1 for the first level and 1.0 for the second level of combination.

Supplementary Figure 1 shows the results from 1000 realisations of simulated data; panels on the left come from Intermediate combination while panels on the right come from Late combination. First, we simulated 1000 “noise” features (drawn from a zero-mean Gaussian with the standard deviation of 1). Panels a1 and a2 show that performance was centred on 50%, as expected. This demonstrates that our cross-validation scheme is unbiased.

Next, we simulated a single feature based on the (Z-scored) MMSE values, as a reference “signal” feature. Panels b1 and b2 show identical mean performance of 73.6% for both Intermediate and Late combination, as expected when only one kernel.

To demonstrate that Early combination is not optimal, we concatenated the 1000 noise features to the MMSE signal feature, and panels c1 and c2 show performance dropped to 54.5%, reflecting the difficulty in finding the signal among so many features.

However, when combining the signal feature and noise features via separate kernels (i.e., 1001 kernels), Panels d1 and d2 show that performance improved related to Early combination, to 58.2% for Intermediate and 70.7% for Late combination. Note the latter is still below the reference levels in Panels b1 and b2 with only the MMSE signal, reflecting imperfect down-weighting of noise kernels in some cases.

Finally, Panels e1 and e2 show the results from 4 “signal” kernels – the original MMSE plus education, age and sex. Performance is now improved to 73.7% for Intermediate combination (Panel e1) and 76.6% for Late combination (Panel e2).

Supplementary Figure 2 shows the distribution of differences between Intermediate and Late combinations. As expected, there is no difference when a single kernel in Panels a, b and c, but there is a reliable improvement (on over 90% of occasions) for Late combination when there are 1001 (Panel d) or 4 (Panel e) kernels.

**
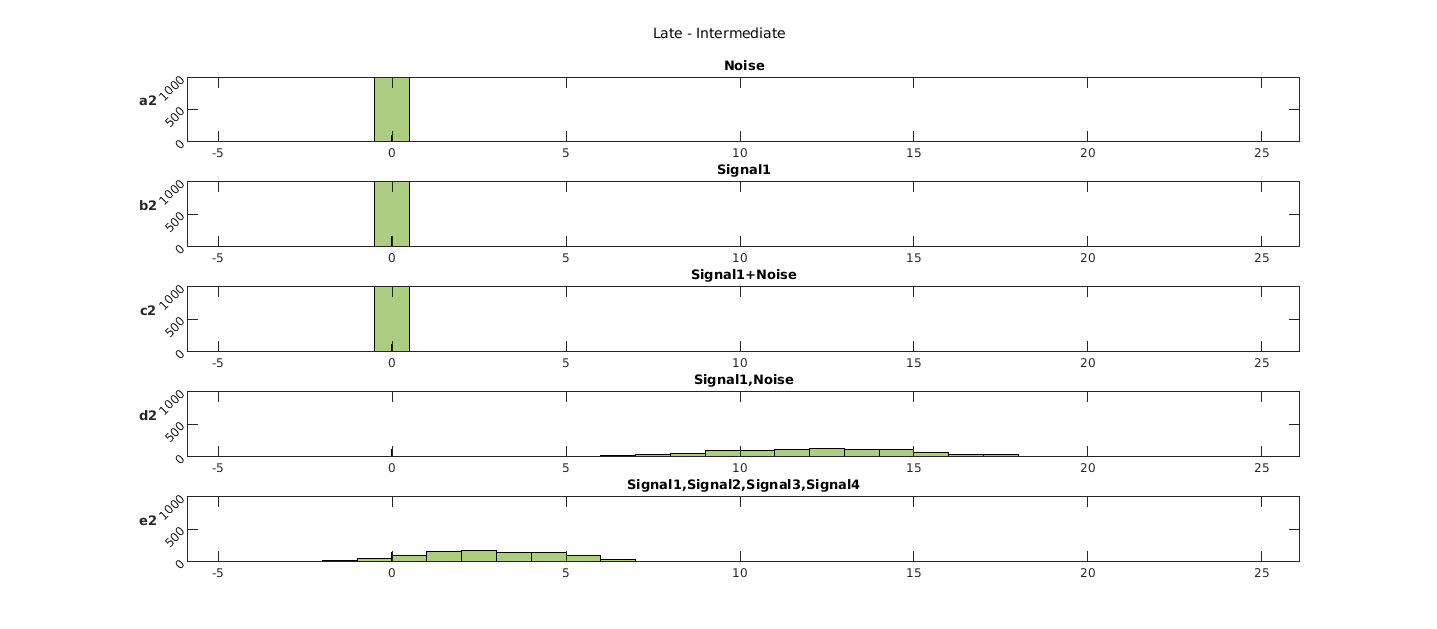
**

**Supplementary Figure 2. Differences in Late versus Intermediate classification accuracies (chance = 0%) from 1000 noise realizations of various kernels (see text). “Noise” refers to 1000 random features drawn from a unit normal distribution; “Signal1” refers to MMSE score; “Signals 2-4” refer to Education, Age and Sex. “A,B” means combining either feature kernels (left column) or prediction kernels (right column) derived from models trained using features A and B.**


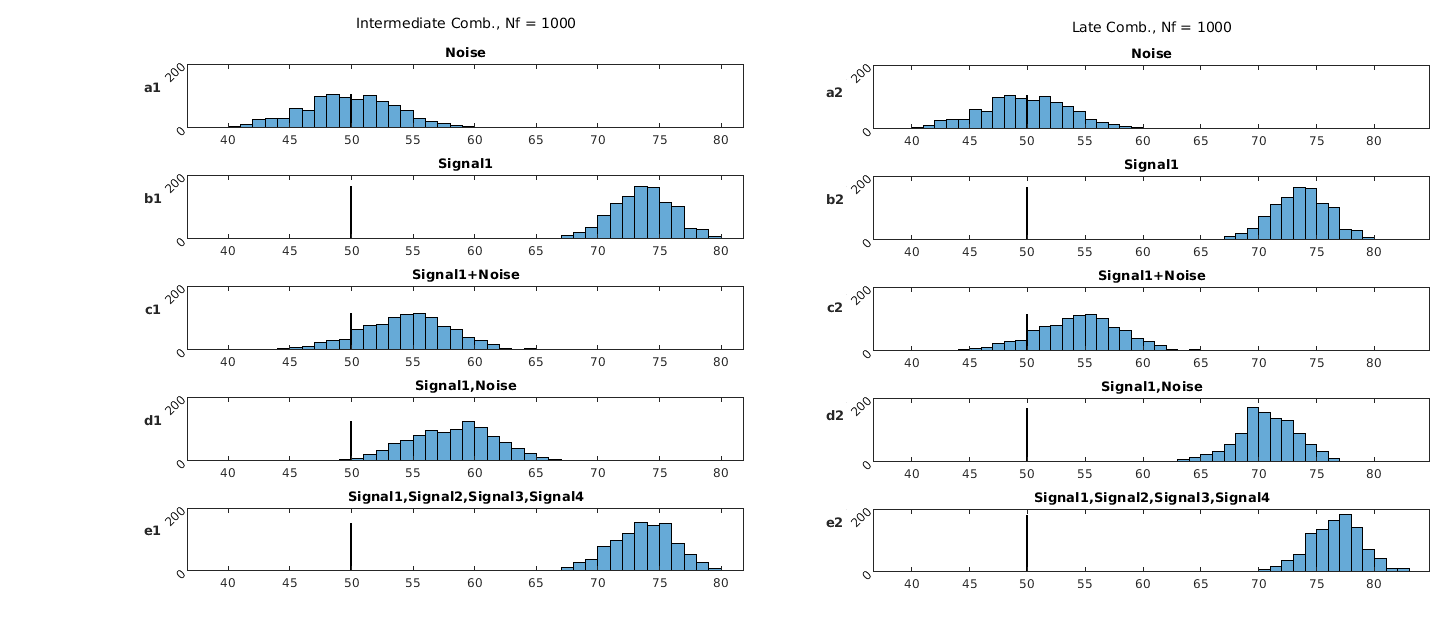


**Supplementary Figure 1 Classification accuracies (chance = 50%) from 1000 noise realizations of various kernels (see text) for Intermediate (left column) and Late (right column) combination. “Noise” refers to 1000 random features drawn from a unit normal distribution; “Signal1” refers to MMSE score; “Signals 2-4” refer to Education, Age and Sex. “A,B” means combining either feature kernels (left column) or prediction kernels (right column) derived from models trained using features A and B; “A+B” means concatenation of features from A and B.**

## S2. Comparison of different modalities using Early or Intermediate combination.

Figure 2 of the main paper shows classification performance for the 8 confounds (COFs), MRI and MEG (covariance of low Gamma in gradiometers) using Late Combination; Supplementary Figures 3 and 4 show the same results for Early and Intermediate combination respectively (note the COFs do not include the MMSE score used Supplementary Figures 1-2, owing to circularity in the MCI classification explained in the main paper).

For Early combination in Panel a1 of Supplementary Figure 3, the concatenation of COFs produced a mean performance of 65.7%, which is slightly above that for Intermediate (Supplementary Figure 4) and Late (main Figure 2) combination using a separate kernel for each confound. The reason for this is considered below. As a result, though MRI still improves classification above COFs on 93.8% of occasions (Panel a2), MEG only improves classification on 79.3% of occasions (Panel b2).


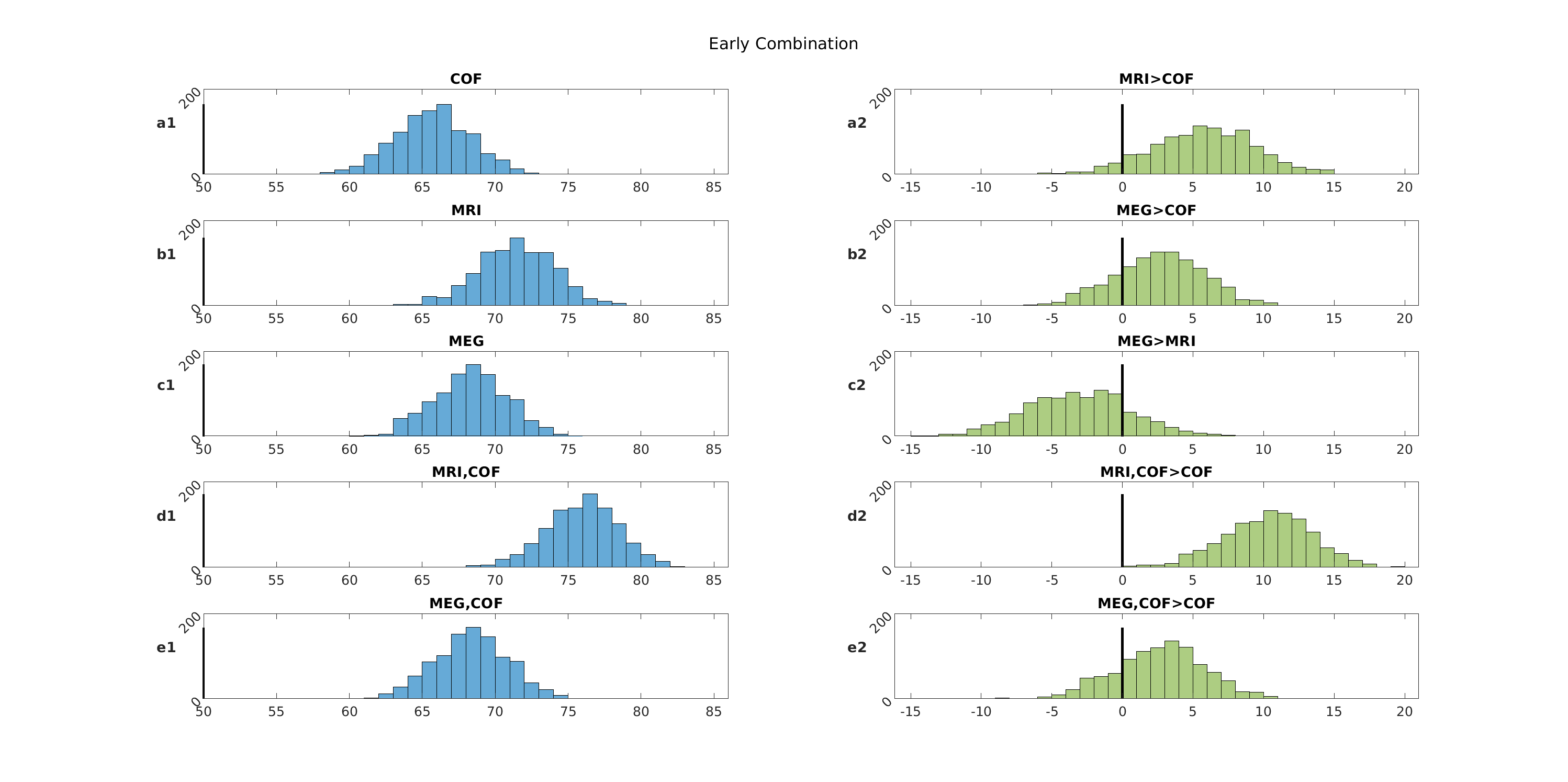


**Supplementary Figure 3 Left column: Classification accuracies (chance = 50%) from 1000 random permutations using Early combinations of MRI, MEG (covariance of gradiometers in low-gamma band) and the 8 potential confounding variables. Right column: Differences in classification performance for each permutation when comparing various combinations of features in left column (where 0 = means no difference). “A,B” means combining two (or nine - in presence of confounds) predictions derived from models trained using modality-type A and modality-type B.**

The results for MRI or MEG alone in Panels b1, c1 and c2 are identical to Intermediate and Late combination because only a single kernel is involved. Early combination of MRI and MEG with COFs shows (Panels d1-e1): adding MRI to COFs still improves classification on 100% of occasions (Panel d2), and adding MEG to COFs only improves on 80.3% of occasions, similar to Intermediate (Supplementary Figure 4) and Late (main Figure 2) combination.

Supplementary Figure 4 shows the same set of comparisons as in Supplementary Figure 3, but now for Intermediate rather than Early combination. As noted above, performance drops slightly to 64.8% for the 8 COFs (Panel a1) relative to Early combination, though is still slightly above that for Late combination (64.5%) in Figure 2 of the main paper. This relative performance for the COFs kernels of Early > Intermediate > Late does not coincide with the case when combining MRI and MEG kernels, as shown in Figure 3 of the main paper, where Late > Intermediate > Early. This demonstrates that Late combination does not always improve over Intermediate combination, and combining kernels (using MKL) does not always improve over simple feature concatenation. The relative advantage of each combination method depends on the number and nature of the features, as well as the regularisation parameters used.


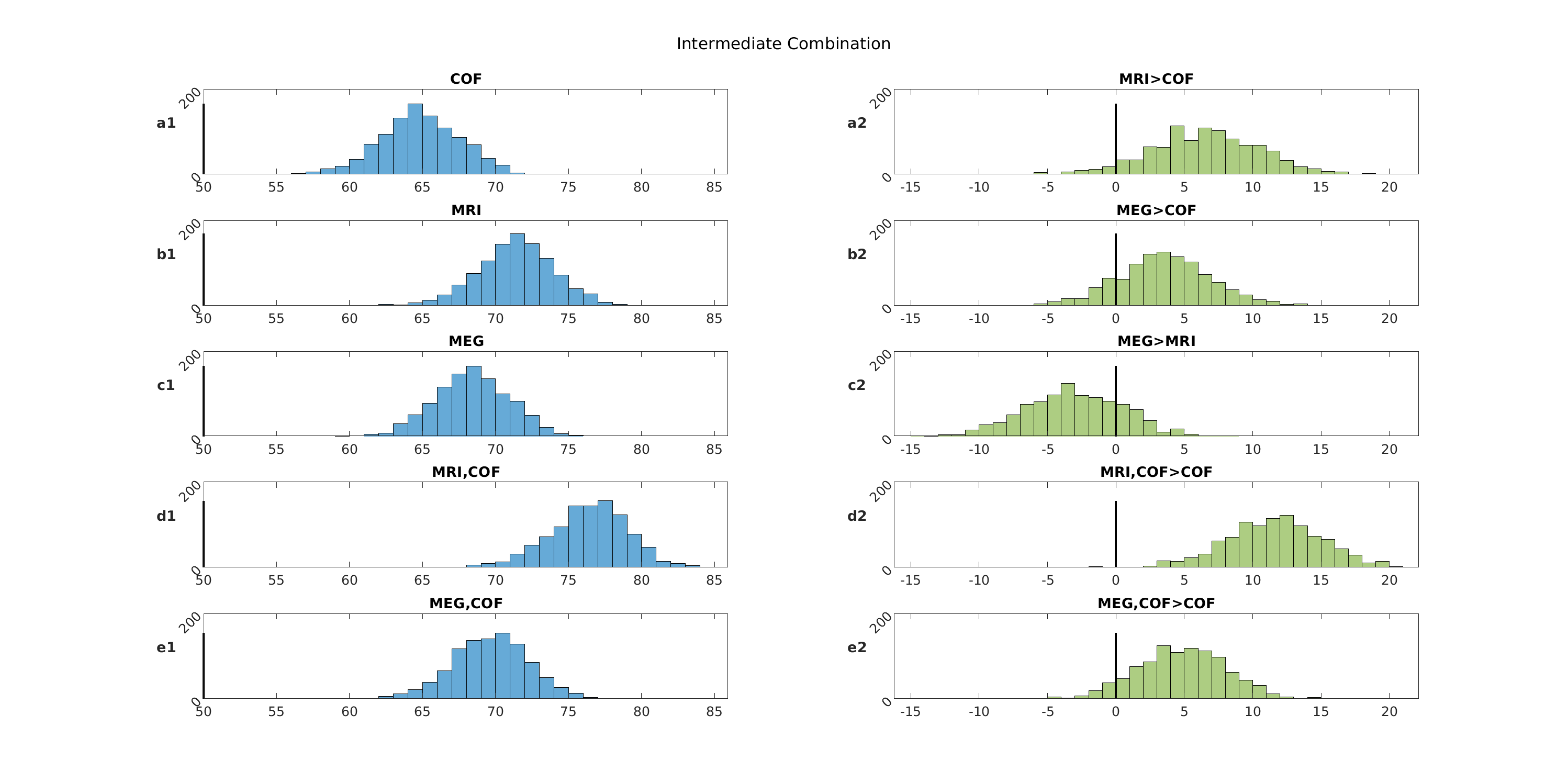


**Supplementary Figure 4 Left column: Classification accuracies (chance = 50%) from 1000 random permutations using Intermediate combinations of MRI, MEG (covariance of gradiometers in low-gamma band) and the 8 potential confounding variables. Right column: Differences in classification performance for each permutation when comparing various combinations of features in left column (where 0 = means no difference). “A,B” means combining two (or nine - in presence of confounds) predictions derived from models trained using modality-type A and modality-type B.**

As above, the combination method has no effect when only one kernel is in Panels b1, c1 and c2. More important is, when combining MEG with COFs (Panel e1), performance is improved relative to Early combination, such that adding MEG to COFs (Panel e2) now does reliably improve classification on over 92.7% of occasions (Panel e2 of Supplementary Figure 3).

## S3. Classification Accuracies for all MEG features

Table 1 in the main paper shows the percentage of occasions when combining MRI with various MEG features improve classification relative to MRI alone; Supplementary Table 1 shows the raw classification accuracies for MEG alone.

| MEG Feature  Frequency band | COV  of MAG | VAR  of MAG | COV  of GRD | VAR  of GRD |
| --- | --- | --- | --- | --- |
| Delta  2-4 Hz | 56.3 (2.9) | 56.0 (2.4) | 60.0 (2.9) | 58.8 (2.6) |
| Theta  4-8 Hz | 59.4 (2.7) | 56.5 (2.6) | 60.0 (2.7) | 58.4 (2.8) |
| Alpha  8-12 Hz | 59.2 (2.8) | 55.6 (2.7) | 59.0 (2.7) | 55.0 (2.7) |
| Beta  12-30 Hz | 63.5 (2.7) | 58.9 (2.9) | 66.6 (2.6) | 64.0 (2.8) |
| Low-Gamma  30-48 Hz | 67.3 (2.5) | 63.4 (2.7) | 68.4 (2.6) | 67.0 (2.6) |
| High-Gamma  52-86 Hz | 67.8 (2.7) | 64.0 (2.8) | 68.2 (2.6) | 66.0 (2.7) |

*Supplementary Table 1. The numbers show mean (and SD in brackets) of classification accuracy from 1000 permutations of 5-fold cross-validation of various MEG features in sensor level.*

Supplementary Figure 5 shows the distribution of classification accuracies across 1000 permutations using Late combination for low gamma band [30-48 Hz], as a function of VAR/COV and MAG/GRD. There was some evidence that covariance classified better than variance (on 88.1% of occasions in Panel a2) and that gradiometers classified better than magnetometers (on 93.4% occasions in Panel c2). There was also suggestive evidence that the information was complementary, in that combining COV with VAR improved classification reliably above VAR alone (Panel b2) on 88.4% of occasions and combining GRD with MAG improved classification reliably above MAG alone on 93.8% of occasions (Panel d2).


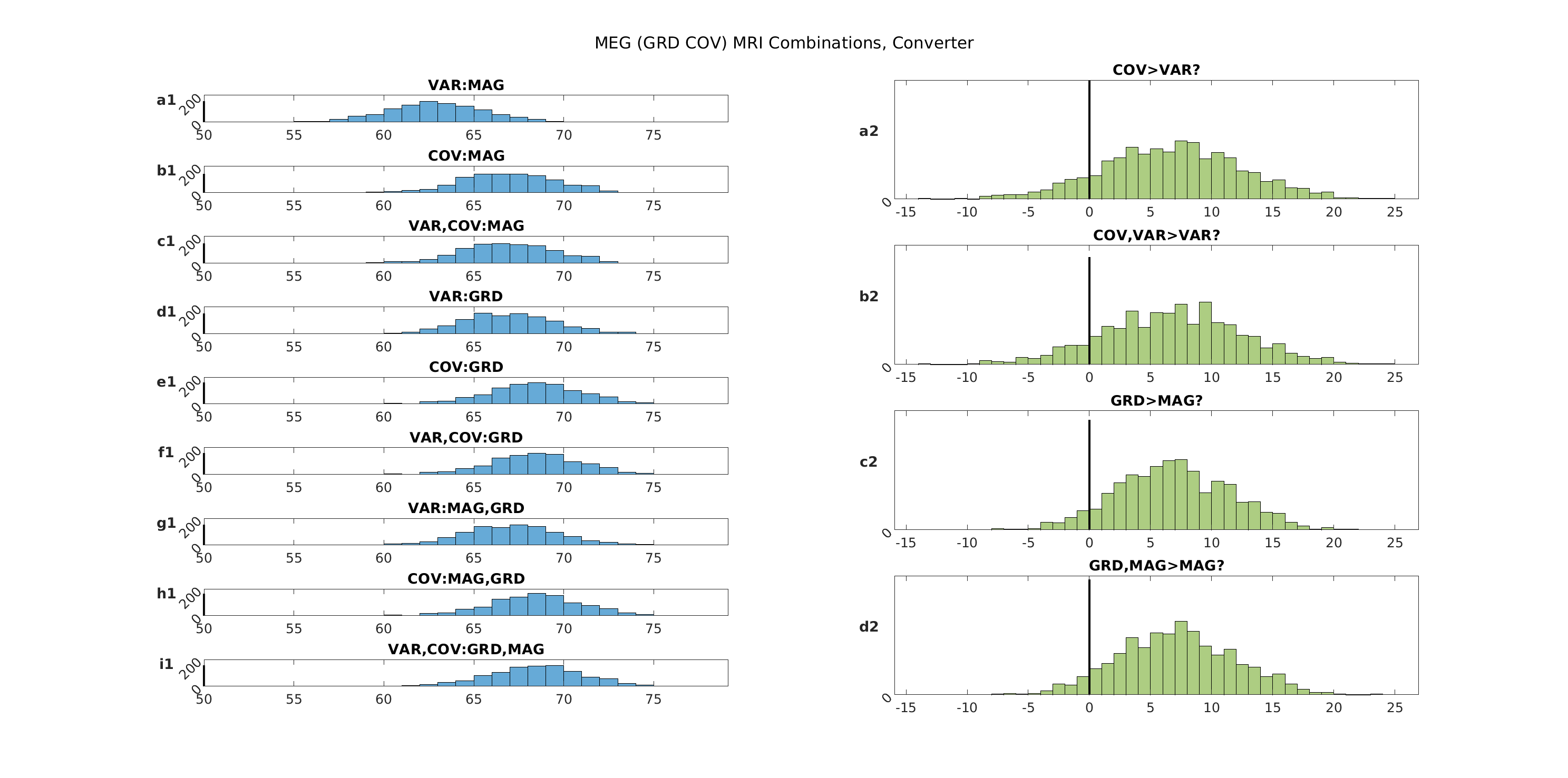


**Supplementary Figure 5 Left column: Classification accuracies (chance = 50%) from 1000 random permutations using Late combinations of different MEG features/sensor. Right column: Differences in classification performance for each permutation when comparing various combinations of features in left column (where 0 = means no difference). “A,B” means combining two predictions derived from models trained using sensor or feature-type A and sensor or feature -type B.**

## S4. Feature selection (reduction) using PCA

Supplementary Figure 6 shows classification accuracies when combining the MRI and MEG features at either early, intermediate or late stages, like Figure 3 in the main paper, but after applying PCA to reduce dimensions to the number needed to explain at least 95% of the variance in the features across participants. Because the PCA was done on the training set only, the number of PCs kept varied. For MEG (covariance of gradiometers in low gamma), it varied between [37-43]; for MRI, it varied between [11-13].

Panel a1 shows that the mean accuracy for Early combination of 69.5% is slightly lower than that without PCA (69.8%). For Intermediate combination, the mean accuracy of 75.0% is slightly better than that without PCA (74.3%) and is improved on Early combination on 93.2% of occasions (Panel a2). The mean accuracy for late combination with PCA is 75.9%, less than the 77.2% without PCA, and improving on Intermediate combination on only 65.6% of occasions (Panel b2). There is also a reduction in performance of MRI alone, from 71.4% without to 69.5% with PCA. Thus, while PCA does not generally help, the important finding is that, even with PCA, late combination of MEG and MRI is still better than MRI alone on 97.7% of occasions (panel c2), and similar results occurred when COFs were added (79.0% of occasions).


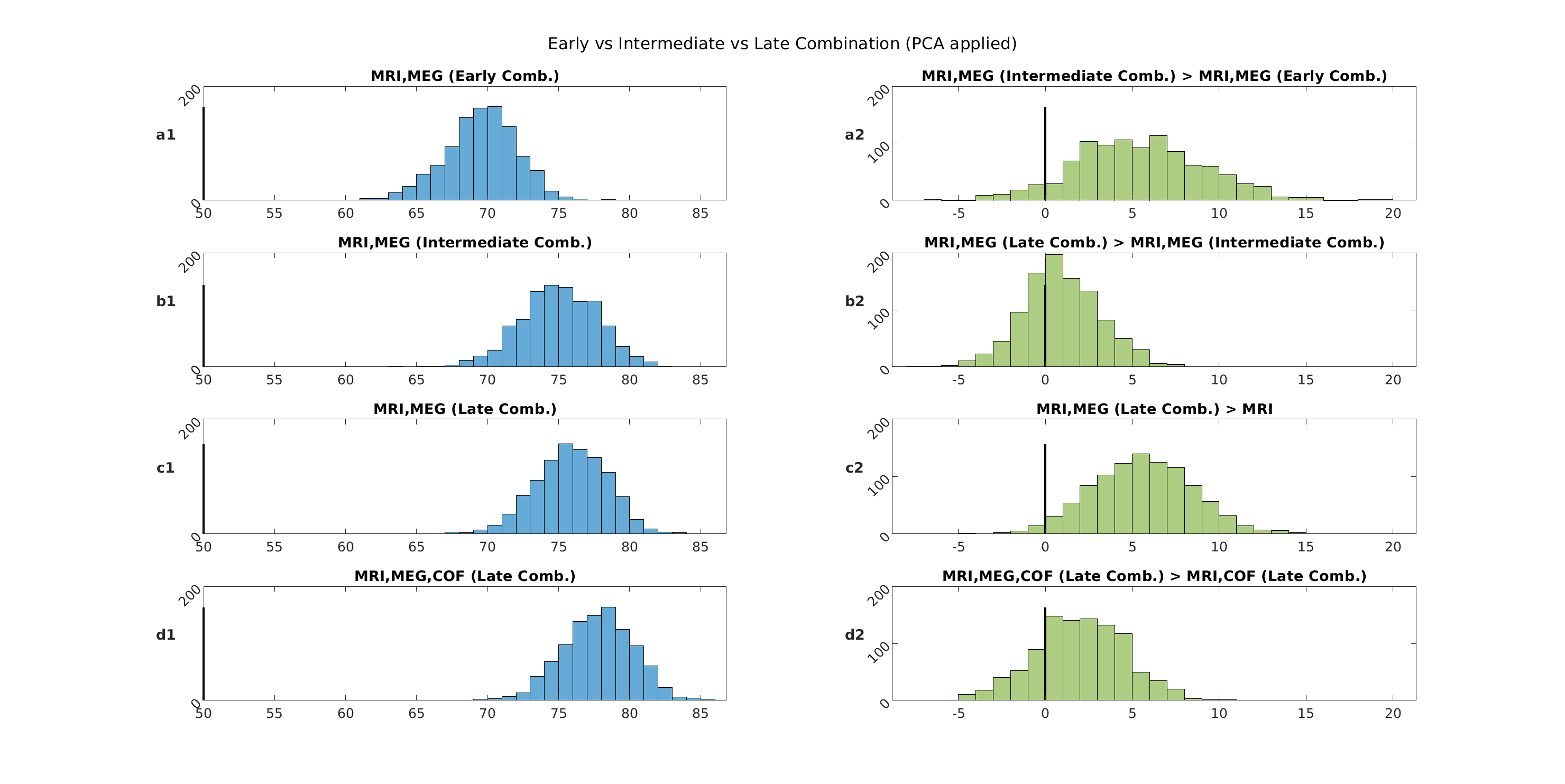


**Supplementary Figure 6 Left column: Classification accuracies (chance = 50%) from 1000 random permutations using MEG, MRI with Early, Intermediate and Late combinations (see Methods), after applying PCA to estimate the number of components within each modality (and each training set) that were needed to explain 95% of the variance across participants. Right column: Corresponding differences in classification performance for each permutation when comparing various combinations approach (where 0 = means no difference).**

**c**

## S5. Classification using other classifiers

Supplementary Figures 7, 8 and 9 show classification accuracies when combining the MRI and MEG features at the late stage using MKL, but using different classifiers in the first stage. Supplementary Figures 7-9 shows the results for KNN, random forest and multi-layer neural network classifiers respectively (see *mkl_ens_n.m* function on paper’s GitHub repository for details of the architecture and parameters of these classifiers). For MRI alone (“a” panels), the mean respective accuracies were 68.9%, 68.3% and 70.6%, which were close, but worse than, the 71.4% using SVM. For MEG (using GRD COV in low gamma band; “b” panels), the respective means were 64.5%, 65.8% and 66.1% respectively, which were again less than SVM (68.4%), which is not surprising, since kernel-based classifiers usually work better when the number of features is much higher than the number of samples. For Late combination of these classifiers (“c” panels), the respective means were 71.1%, 72.0% and 75.1% respectively, again worse than the SVM figure of 77.2%. Thus, while we cannot claim the SVM classifier used in our main MKL results is the best possible approach, it does better than three other common classifiers.


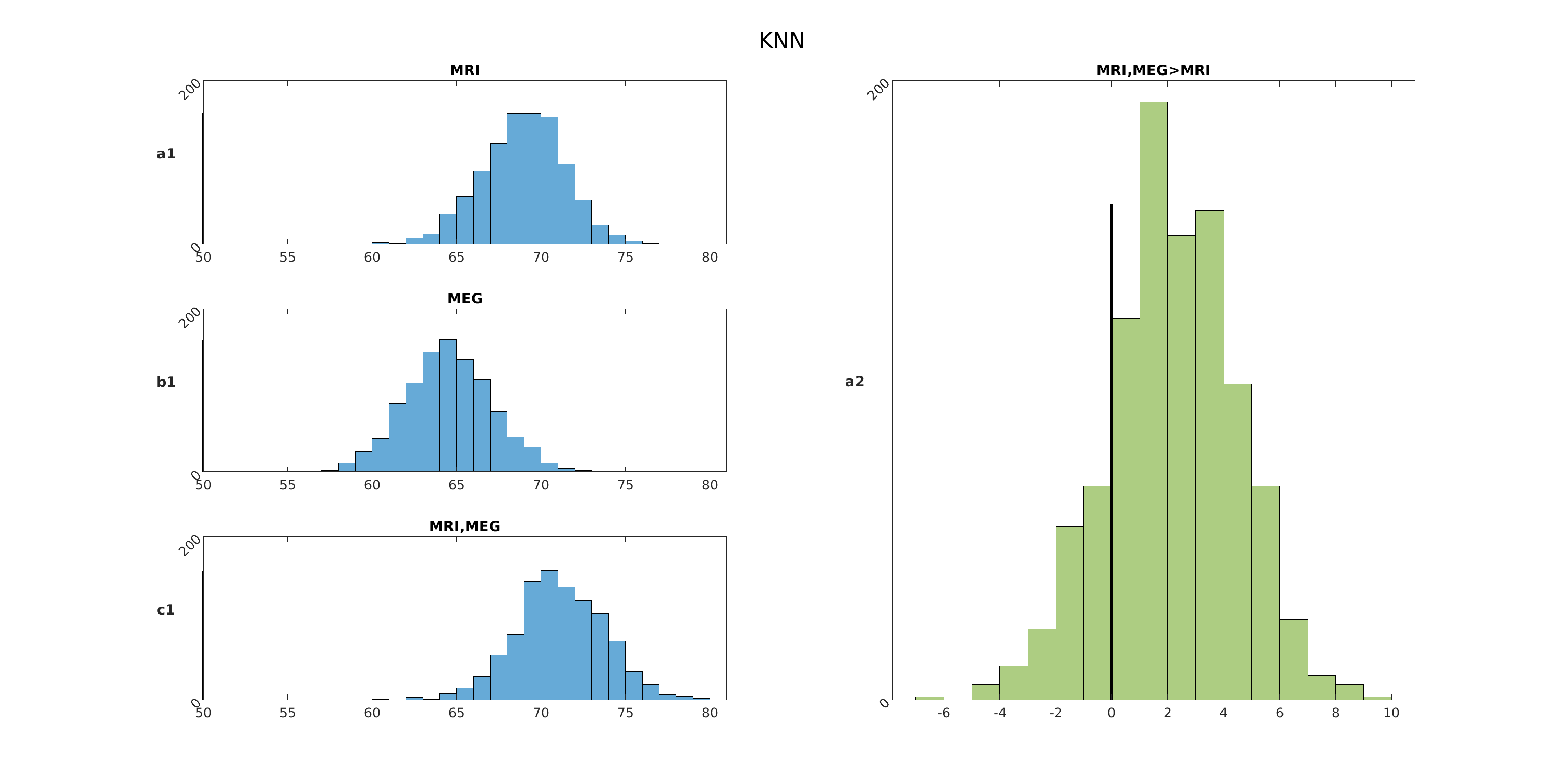


**Supplementary Figure 7 Left column: Classification accuracies (chance = 50%) from 1000 random permutations using MEG, MRI with Late combination where the first stage classifiers are K-nearest neighbors (KNN). Right column: Corresponding differences in classification performance for each permutation when comparing different modalities (where 0 = means no difference).**


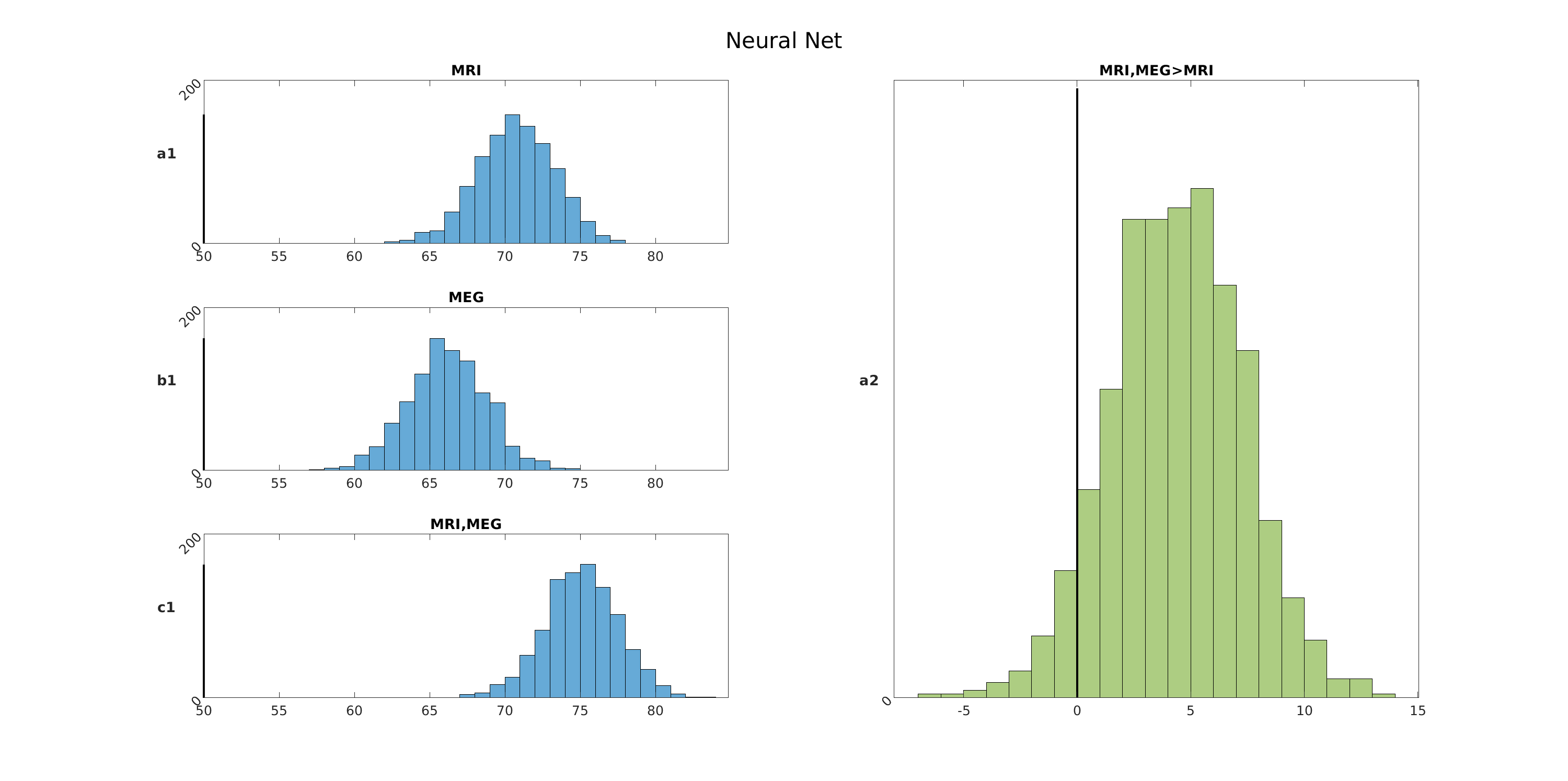


**Supplementary Figure 9 Left column: Classification accuracies (chance = 50%) from 1000 random permutations using MEG, MRI with Late combination where the first stage classifiers are Neural Networks. Right column: Corresponding differences in classification performance for each permutation when comparing different modalities (where 0 = means no difference).**


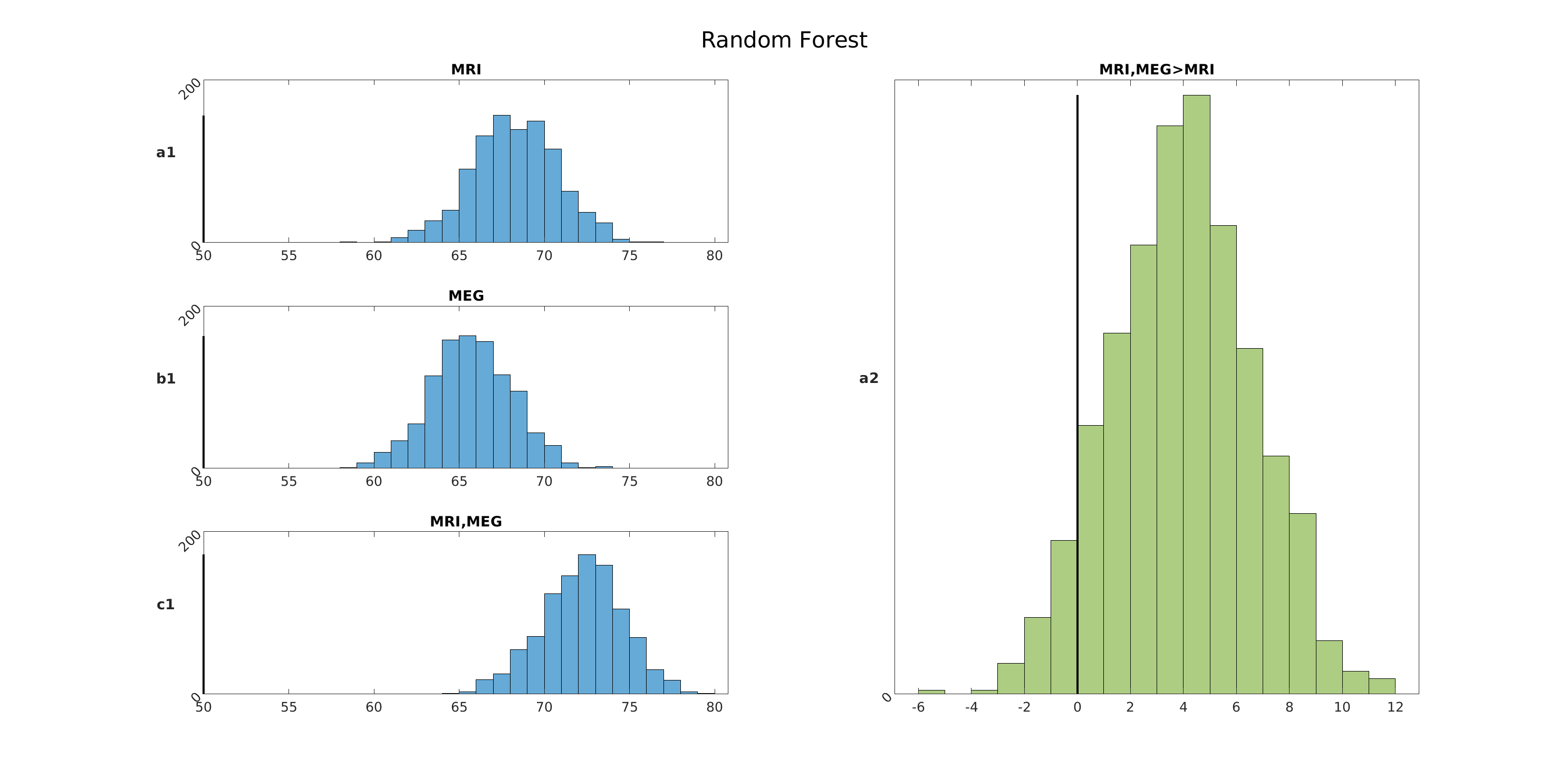


**Supplementary Figure 8 Left column: Classification accuracies (chance = 50%) from 1000 random permutations using MEG, MRI with Late combination where the first stage classifiers are Random Forest. Right column: Corresponding differences in classification performance for each permutation when comparing different modalities (where 0 = means no difference).**

However, our main aim is not to find the best classifier, but to check whether the claim that MEG adds to MRI in MCI classification generalizes across more than one classifier. This appears to be the case in the “d” panels of Supplementary Figures 7-9, which show improvements for 83.5%, 92.1% and 94.1% of occasions, for KNN, random forest and neural networks respectively (cf. 97.5% when using SVM MKL in the main text). Thus, there is nearly always a significant improvement, with the percentage of improved classifications itself increasing as overall accuracy increased.

## S6. Classification using all MRI voxels

In the main paper, the MRI images were reduced to 110 features using the Harvard Oxford Atlas (HOA). It is possible that these ROIs are too coarse to capture early effects of AD (e.g., in anterior versus posterior parts of the hippocampal ROI). We, therefore, repeated analyses using the local GM estimate for every GM voxel in the MRIs. This resulted in 390,189 voxels that were non-zero for every participant. Note that this is now an order of magnitude larger than the number of MEG features (20,706 for GRD COV), as opposed to being two orders of magnitude smaller when using the 110 HOA ROIs in the main paper.

Panel a1 in Supplementary Figure 10 shows the distribution across 1000 permutations of classification accuracies based on one kernel for MRI voxel features. The mean accuracy was 72.4%, above chance (50%) on 100% of occasions, and slightly better than the ROI data (71.4%). Panel c1 of Supplementary Figure 10 shows classification accuracies when combining the MRI and MEG features using MKL. The mean accuracy is 74.4% which is still more than MRI alone (72.4%). Most importantly, panel a2 shows MEG and MRI improve classification accuracy compared to MRI alone on nearly 93.7% of occasions, which is less often than with the ROI data in the main paper (97.5%).


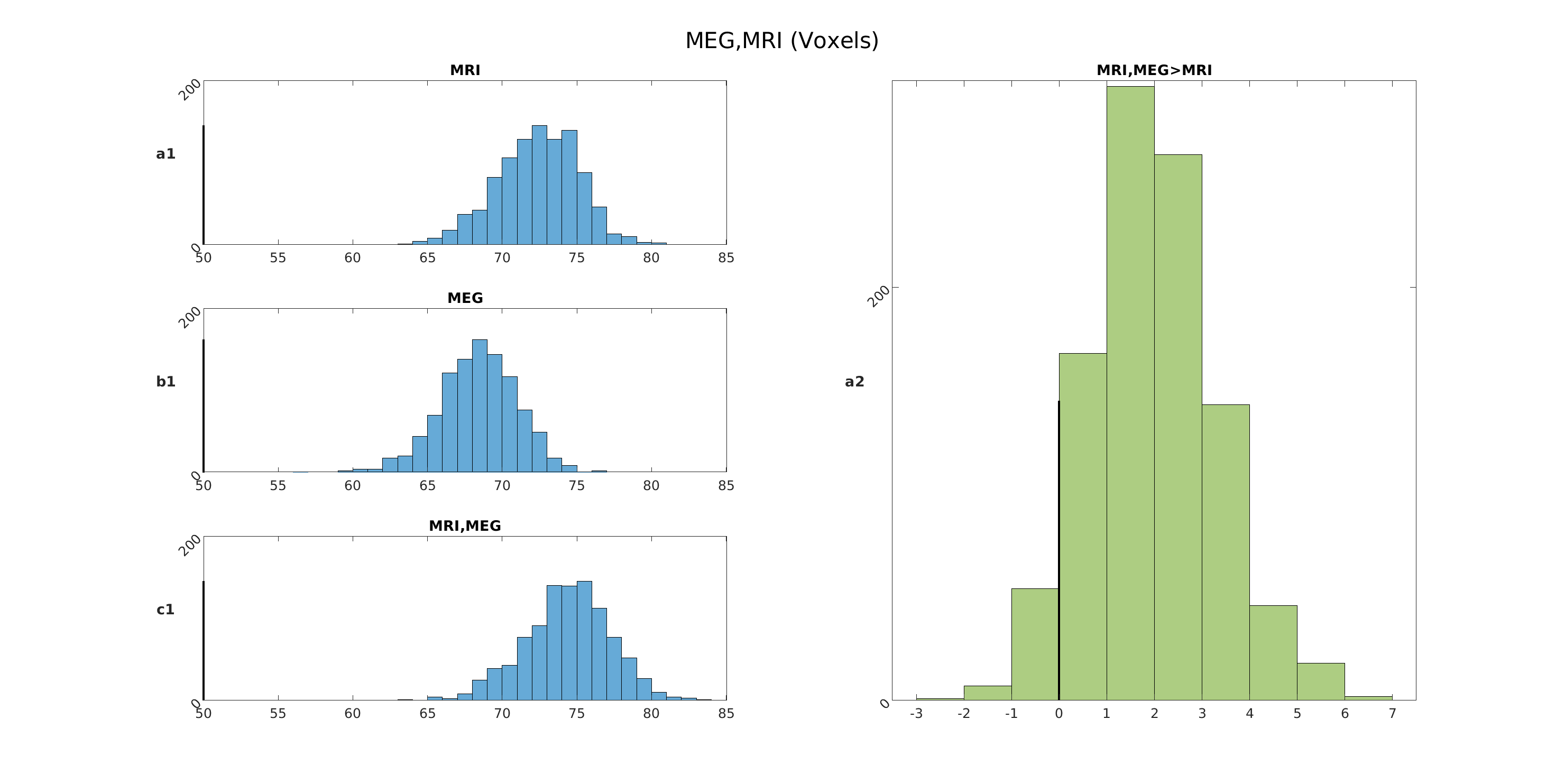


**Supplementary Figure 10 Left column: Classification accuracies (chance = 50%) from 1000 random permutations using MEG, MRI with MKL where the features for MRI includes all GM voxels. Right column: Corresponding differences in classification performance for each permutation when comparing different modalities (where 0 = means no difference).**

## S7. Combining MRI and MEG features in classifying converter vs non-converter MCIs

For a subset of individuals with MCI (i.e., 106 of the 144 cases), follow-up data are available that show that 55 later converted into “probable AD”, while 51 remained stable (non-converters). Given that MCI is well-known to have a range of aetiologies beyond neurodegeneration, the classification of converters versus non-converters is potentially a more sensitive indicator of AD. We, therefore, explored all 24 possibilities of variance/covariance (VAR/COV), gradiometers/magnetometers (GRD/MAG) and the 6 frequency bands for classifying MCI converters versus MCI non-converters (using Late combination). The results are shown in Supplementary Table 3.

| MEG Feature  Frequency band | COV  of MAG | VAR  of MAG | COV  of GRD | VAR  of GRD |
| --- | --- | --- | --- | --- |
| Delta  2-4 Hz | 70.9 (4.8)  82.2%  +2.18 | 70.1 (5.0)  69.6%  +1.35 | 71.8 (5.0)  82.3%  +3.05 | 69.7 (5.0)  62.5%  +0.91 |
| Theta  4-8 Hz | 71.2 (4.6)  74.9%  +2.48 | 70.1 (4.9)  71.0%  +1.35 | 71.4 (4.8)  74.5%  +2.68 | 69.6 (4.9)  62.00%  +0.81 |
| Alpha  8-12 Hz | 69.9 (4.9)  68.8%  +1.18 | 69.8 (5.0)  67.2%  +1.10 | 70.2 (5.0)  66.9%  +1.52 | 69.7 (5.0)  65.4%  +0.95 |
| Beta  12-30 Hz | 69.5 (4.9)  64.2%  +0.71 | 69.0 (5.0)  54.6%  +0.29 | 69.5 (5.1)  58.0%  +0.77 | 69.4 (5.0)  59.4%  +0.70 |
| Low-Gamma  30-48 Hz | 68.4 (4.7)  46.3%  -0.33 | 68.6 (4.9)  44.4%  -0.10 | 69.07 (4.8)  51.5%  +0.36 | 69.9 (4.9)  65.1%  +1.14 |
| High-Gamma  52-86 Hz | 70.2 (4.6)  67.1%  +1.42 | 69.7 (4.9)  62.4%  +0.99 | 69.58 (4.8)  57.6%  +0.87 | 70.3 (5.0)  65.0%  +1.55 |

*Supplementary Table 2 Exploring the MEG feature space for converters vs non-converters. The top numbers show mean (and SD in brackets) of classification accuracy when combining the relevant MEG feature with MRI; the middle number shows the percentage of permutations in which this accuracy exceeded that of MRI alone (68.7%), where chance = 50%; the bottom number shows* the average difference between MEG-MRI combination and MRI alone.

Classification accuracies were generally lower than in Table 2, most likely because of the much-reduced sample size (from 307 to 106). Classification performance using MRI alone was 68.7%. Interestingly, it was now the lower frequency bands (particularly delta and theta) that tended to show an improvement when combined with MRI, relative to MRI alone (and again the COV of GRD that seemed to provide the best MEG features). Though no single MEG feature set showed a highly reliable improvement when combined with MRI (i.e., >90%), again most likely due to the reduced sample size, there was a consistent trend for an improvement in the vast majority of cases.

## S8. Optimised Hyper-parameters

| **Feature** | **Type** | **λ1** | **λ2** |
| --- | --- | --- | --- |
| COF | N/A | 0.57 | n/a |
| [COF] | N/A | 0.43 | n/a |
| COF (8 Kernels) | N/A | 0.01 | 0.60 |
| MRI | Voxels | 0.22 | n/a |
| MRI | ROI data | 0.16 | n/a |
| MRI,COF (8 Kernels) | ROI data | 0.18 | 0.53 |
| MEG,COF (8 Kernels) | GRD COV (Gamma1) | 0.09 | 0.70 |
| MRI,MEG,COF(8 Kernels) | GRD COV (Gamma1),ROI data | 0.15 | 0.67 |
| COF | PCA applied | 0.50 | n/a |
| MEG | GRD COV (Gamma1) - PCA applied | 0.37 | n/a |
| MRI | ROI data - PCA applied | 0.20 | n/a |
| MRI,MEG | GRD COV (Gamma1), ROI data -PCA applied | 0.14 | 0.62 |
| MRI,COF (8 Kernels) | PCA applied | 0.11 | 0.44 |
| MRI,MEG,COF (8 Kernels) | PCA applied | 0.14 | 0.62 |
| [MRI MEG] | PCA applied | 0.38 | n/a |
| [MRI MEG COF] | PCA applied | 0.40 | n/a |
| MEG | MAG COV (Delta) | 0.54 | n/a |
| MEG | MAG COV (Theta) | 0.42 | n/a |
| MEG | MAG COV (Alpha) | 0.41 | n/a |
| MEG | MAG COV (Beta) | 0.34 | n/a |
| MEG | MAG COV (Gamma1) | 0.27 | n/a |
| MEG | MAG COV (Gamma2) | 0.33 | n/a |
| MEG | MAG VAR (Delta) | 0.66 | n/a |
| MEG | MAG VAR (Theta) | 0.63 | n/a |
| MEG | MAG VAR (Alpha) | 0.47 | n/a |
| MEG | MAG VAR (Beta) | 0.34 | n/a |
| MEG | MAG VAR (Gamma1) | 0.27 | n/a |
| MEG | MAG VAR (Gamma2) | 0.33 | n/a |
| MEG | GRD COV (Delta) | 0.16 | n/a |
| MEG | GRD COV (Theta) | 0.29 | n/a |
| MEG | GRD COV (Alpha) | 0.29 | n/a |
| MEG | GRD COV (Beta) | 0.31 | n/a |
| MEG | GRD COV (Gamma1) | 0.40 | n/a |
| MEG | GRD COV (Gamma2) | 0.44 | n/a |
| MEG | GRD VAR (Delta) | 0.28 | n/a |
| MEG | GRD VAR (Theta) | 0.34 | n/a |
| MEG | GRD VAR (Alpha) | 0.32 | n/a |
| MEG | GRD VAR (Beta) | 0.31 | n/a |
| MEG | GRD VAR (Gamma1) | 0.21 | n/a |
| MEG | GRD VAR (Gamma2) | 0.29 | n/a |
| MRI,MEG | MAG COV (Delta) | 0.41 | 0.36 |
| MRI,MEG | MAG COV (Theta) | 0.24 | 0.38 |
| MRI,MEG | MAG COV (Alpha) | 0.17 | 0.59 |
| MRI,MEG | MAG COV (Beta) | 0.20 | 0.65 |
| MRI,MEG | MAG COV (Gamma1) | 0.14 | 0.54 |
| MRI,MEG | MAG COV (Gamma2) | 0.13 | 0.75 |
| MRI,MEG | MAG VAR (Delta) | 0.49 | 0.38 |
| MRI,MEG | MAG VAR (Theta) | 0.48 | 0.40 |
| MRI,MEG | MAG VAR (Alpha) | 0.24 | 0.60 |
| MRI,MEG | MAG VAR (Beta) | 0.24 | 0.61 |
| MRI,MEG | MAG VAR (Gamma1) | 0.21 | 0.50 |
| MRI,MEG | MAG VAR (Gamma2) | 0.27 | 0.45 |
| MRI,MEG | GRD COV (Delta) | 0.30 | 0.39 |
| MRI,MEG | GRD COV (Theta) | 0.22 | 0.45 |
| MRI,MEG | GRD COV (Alpha) | 0.17 | 0.63 |
| MRI,MEG | GRD COV (Beta) | 0.13 | 0.71 |
| MRI,MEG | GRD COV (Gamma1) | 0.15 | 0.74 |
| MRI,MEG | GRD COV (Gamma2) | 0.13 | 0.52 |
| MRI,MEG | GRD VAR (Delta) | 0.39 | 0.36 |
| MRI,MEG | GRD VAR (Theta) | 0.40 | 0.43 |
| MRI,MEG | GRD VAR (Alpha) | 0.20 | 0.63 |
| MRI,MEG | GRD VAR (Beta) | 0.20 | 0.63 |
| MRI,MEG | GRD VAR (Gamma1) | 0.14 | 0.52 |
| MRI,MEG | GRD VAR (Gamma2) | 0.15 | 0.51 |
| [MRI COF] | ROI data | 0.15 | n/a |
| [MEG COF] | GRD COV (Gamma1) | 0.40 | n/a |
| [MRI MEG] | GRD COV (Gamma1),ROI data | 0.30 | n/a |
| [MRI MEG COF] | GRD COV (Gamma1),ROI data | 0.35 | n/a |
| VAR(MAG),COV(MAG) | (Gamma1) | 0.02 | 0.72 |
| VAR(GRD),COV(GRD) | (Gamma1) | 0.12 | 0.64 |
| VAR(GRD),VAR(MAG) | (Gamma1) | 0.10 | 0.50 |
| COV(GRD),COV(MAG) | (Gamma1) | 0.07 | 0.52 |
| VAR(MAG),VAR(GRD),COV(MAG),COV(GRD) | (Gamma1) | 0.13 | 0.60 |
| MRI,MEG | GRD COV (Gamma1),Voxels | 0.14 | 0.55 |
| MRI (converters) | ROI data | 0.38 | n/a |
| MEG (converters) | MAG COV (Delta) | 0.45 | n/a |
| MEG (converters) | MAG COV (Theta) | 0.24 | n/a |
| MEG (converters) | MAG COV (Alpha) | 0.56 | n/a |
| MEG (converters) | MAG COV (Beta) | 0.54 | n/a |
| MEG (converters) | MAG COV (Gamma1) | 0.46 | n/a |
| MEG (converters) | MAG COV (Gamma2) | 0.49 | n/a |
| MEG (converters) | MAG VAR (Delta) | 0.28 | n/a |
| MEG (converters) | MAG VAR (Theta) | 0.36 | n/a |
| MEG (converters) | MAG VAR (Alpha) | 0.48 | n/a |
| MEG (converters) | MAG VAR (Beta) | 0.52 | n/a |
| MEG (converters) | MAG VAR (Gamma1) | 0.59 | n/a |
| MEG (converters) | MAG VAR (Gamma2) | 0.46 | n/a |
| MEG (converters) | GRD COV (Delta) | 0.45 | n/a |
| MEG (converters) | GRD COV (Theta) | 0.33 | n/a |
| MEG (converters) | GRD COV (Alpha) | 0.61 | n/a |
| MEG (converters) | GRD COV (Beta) | 0.60 | n/a |
| MEG (converters) | GRD COV (Gamma1) | 0.62 | n/a |
| MEG (converters) | GRD COV (Gamma2) | 0.60 | n/a |
| MEG (converters) | GRD VAR (Delta) | 0.27 | n/a |
| MEG (converters) | GRD VAR (Theta) | 0.43 | n/a |
| MEG (converters) | GRD VAR (Alpha) | 0.61 | n/a |
| MEG (converters) | GRD VAR (Beta) | 0.52 | n/a |
| MEG (converters) | GRD VAR (Gamma1) | 0.58 | n/a |
| MEG (converters) | GRD VAR (Gamma2) | 0.50 | n/a |
| MRI,MEG (converters) | MAG COV (Delta) | 0.12 | 0.52 |
| MRI,MEG (converters) | MAG COV (Theta) | 0.15 | 0.37 |
| MRI,MEG (converters) | MAG COV (Alpha) | 0.07 | 0.63 |
| MRI,MEG (converters) | MAG COV (Beta) | 0.09 | 0.40 |
| MRI,MEG (converters) | MAG COV (Gamma1) | 0.07 | 0.64 |
| MRI,MEG (converters) | MAG COV (Gamma2) | 0.13 | 0.53 |
| MRI,MEG (converters) | MAG VAR (Delta) | 0.33 | 0.33 |
| MRI,MEG (converters) | MAG VAR (Theta) | 0.26 | 0.26 |
| MRI,MEG (converters) | MAG VAR (Alpha) | 0.44 | 0.44 |
| MRI,MEG (converters) | MAG VAR (Beta) | 0.36 | 0.36 |
| MRI,MEG (converters) | MAG VAR (Gamma1) | 0.30 | 0.30 |
| MRI,MEG (converters) | MAG VAR (Gamma2) | 0.30 | 0.30 |
| MRI,MEG (converters) | GRD COV (Delta) | 0.15 | 0.53 |
| MRI,MEG (converters) | GRD COV (Theta) | 0.07 | 0.58 |
| MRI,MEG (converters) | GRD COV (Alpha) | 0.12 | 0.67 |
| MRI,MEG (converters) | GRD COV (Beta) | 0.02 | 0.35 |
| MRI,MEG (converters) | GRD COV (Gamma1) | 0.07 | 0.49 |
| MRI,MEG (converters) | GRD COV (Gamma2) | 0.02 | 0.30 |
| MRI,MEG (converters) | GRD VAR (Delta) | 0.14 | 0.38 |
| MRI,MEG (converters) | GRD VAR (Theta) | 0.16 | 0.34 |
| MRI,MEG (converters) | GRD VAR (Alpha) | 0.13 | 0.53 |
| MRI,MEG (converters) | GRD VAR (Beta) | 0.07 | 0.34 |
| MRI,MEG (converters) | GRD VAR (Gamma1) | 0.05 | 0.35 |
| MRI,MEG (converters) | GRD VAR (Gamma2) | 0.08 | 0.30 |

*Supplementary Table 3: Optimized hyper parameters for both first stage of feature kernel combination and the second stage of decision combination using ensemble nested cross-validation of various features used in analyses. “[A B]” means concatenation of features from modality-type A and modality-type B. “A,B” means combining two (or nine - in presence of confounds) predictions derived from models trained using modality-type A and modality-type B.*
